# Spontaneous Modulation of Standard EEG Frequency Bands During a Neurofeedback‐Like Task

**DOI:** 10.1111/psyp.70163

**Published:** 2025-10-04

**Authors:** Jacob Maaz, Véronique Paban, Laurent Waroquier, Arnaud Rey

**Affiliations:** ^1^ Aix‐Marseille Université, CNRS, CRPN Marseille France; ^2^ Institute Neuro‐Marseille, Aix‐Marseille Université Marseille France; ^3^ Institute of Language, Communication and the Brain, Aix‐Marseille Université Marseille France; ^4^ Aix‐Marseille Université, PSYCLE Aix‐en‐Provence France

**Keywords:** EEG, neurofeedback, neuromodulation, spectral power

## Abstract

Widely used to treat cognitive, affective, psychiatric, and neurological disorders, electroencephalographic neurofeedback (EEG‐NF) provides individuals with real‐time feedback of their EEG activity to modify brain function. However, the mechanisms behind the EEG changes targeted by EEG‐NF remain unclear. The present study addresses this gap by examining methodological issues in the assessment of spontaneous EEG changes during EEG‐NF sessions. Over multiple trials, healthy young adults observed a gray circle that either remained constant (control condition) or was continuously modified in size at different frequency rates (1, 5, and 10 Hz). We investigated whether EEG frequency bands classically targeted by EEG‐NF: (i) change spontaneously over time, (ii) are influenced by a continuously modified visual stimulus, and (iii) the frequency at which this stimulus is modified. Results revealed: (i) a spontaneous increase in alpha power throughout the entire task, (ii) an increase in theta power when exposing participants to a continuous modification of the visual stimulus (relative to perceiving the same unmodified stimulus), and (iii) an absence of changes in the EEG frequency bands studied when manipulating the frequency of stimulus modification. These findings suggest that the EEG changes observed during EEG‐NF are influenced by the task environment itself and not only by successful EEG self‐modulation. It is therefore crucial to carefully design EEG‐NF protocols to account for non‐specific effects and ensure that observed EEG changes are due to the hypothesized mechanisms. Further research is needed to delineate the mechanisms underlying EEG modulation in EEG‐NF and to refine protocols prior to clinical application.

## Impact Statement

This study adds a new perspective on the mechanisms responsible for the EEG modulation reported in EEG neurofeedback studies. While it is historically claimed that spectral changes within classic EEG frequency bands are due to an active self‐regulation mechanism, we highlight that these changes may be partly due to uncontrolled factors intrinsic to neurofeedback protocols (spontaneous fluctuations, processing of visual information).

## 1 Introduction

Electroencephalographic Neurofeedback (EEG‐NF) typically involves humans in a self‐regulation task during which they receive a real‐time external feedback of their own EEG activity (Sitaram et al. 2017). By modulating the EEG power of frequency bands underlying specific pathologies, this technique stands as an effective treatment for a wide range of cognitive, affective, psychiatric and neurological disorders (Arns et al. 2017; Micoulaud‐Franchi et al. 2015; Thibault et al. 2016). For instance, EEG‐NF is effective in treating depression and anxiety disorders (Hammond 2005), attentional deficit hyperactivity disorders (ADHD; Enriquez‐Geppert et al. 2019) or pharmacoresistant epilepsy (Tan et al. 2009). However, despite its increasing popularity among academics and medical practitioners, the clinical relevance of EEG‐NF remains in a decade‐long debate (Kalokairinou, Sullivan, and Wexler 2022; Loo and Makeig 2012; Thibault et al. 2015). Recent publications of rigorously designed studies have brought to light concerns regarding its effectiveness on clinical outcomes (Neurofeedback Collaborative Group 2021, 2023; Schabus et al. 2017; Schönenberg et al. 2017). The clinical benefits, while indeed present, may actually be driven by non‐specific factors such as placebo effects, sustained concentration over iterative sessions, interactions with practitioners, as well as patients' expectations and motivation levels (Schönenberg et al. 2021; Thibault et al. 2017; Thibault and Raz 2017). In parallel, the growing use of EEG‐NF to enhance behavioral performance in healthy subjects is also beginning to face scrutiny regarding its actual effectiveness (Dessy et al. 2018).

To date, the field has mainly focused on establishing that EEG‐NF protocols are effective treatments for various disorders (Thibault et al. 2018). The clinical benefits of EEG‐NF are classically attributed to the modulation of EEG power within specific frequency bands that are directly related to the targeted clinical outcomes (the so‐called “neurophysiological mechanisms”; Micoulaud‐Franchi et al. 2019). Changes in EEG power are typically expected to occur across EEG‐NF sessions, across the trials of a single session, as well as between pre‐ and post‐intervention resting‐state EEG activity (Ros et al. 2020). Yet, the mechanisms underlying each of these three forms of EEG modulation through EEG‐NF are still poorly understood (Micoulaud‐Franchi and Fovet 2018; Pigott et al. 2017, 2018). For instance, resting‐state EEG is not systematically modulated (Neurofeedback Collaborative Group 2021; Schabus et al. 2017; Schönenberg et al. 2017), and discrepancies are sparked over the relevant outcome to measure the success of the EEG modulation, necessary to expect specific clinical benefits (Mirifar et al. 2022; Schabus 2017, 2018; Witte et al. 2018). Importantly, basic interrogations persist regarding the natural variability of targeted frequency bands in EEG‐NF settings (Witte et al. 2018).

Therefore, it remains unclear whether EEG changes observed during EEG‐NF solely result from an active modulating mechanism or are influenced by confounding factors inherent to EEG‐NF tasks (e.g., sustained attention to the variations of the same stimuli over iterative periods). Especially, there is a critical lack of standardization in EEG‐NF protocols (Gruzelier 2014; Strehl 2014). Indeed, significant differences between protocols include the frequency and number of sessions, the number of trials per session, the duration of trials and the type of stimuli used as feedback (Chiasson et al. 2023; Hasslinger et al. 2022). As a result, a spurious environment in EEG‐NF studies steadily fails to optimally induce the desired EEG modulation. Thus, before diving to clinical applications, more fundamental, basic studies in healthy individuals are required to develop a better understanding of EEG modulation through EEG‐NF (Chiasson et al. 2023; Witte et al. 2018).

In line with these existing challenges and uncertainties, we submitted healthy young adults to a passive task mimicking the time structure and environment of an EEG‐NF session, albeit without involving them into a genuine EEG‐NF task (i.e., controlling one's own brain activity). The aim was to evaluate whether the time structure and the feedback presentation of an EEG‐NF session influence the EEG power of classically targeted frequency bands, that is, theta (4–8 Hz), alpha (8–12 Hz), sensorimotor rhythm (SMR, 12–15 Hz), and beta (15–30 Hz) (Dessy et al. 2020; Micoulaud‐Franchi et al. 2015, 2019; Thibault et al. 2015). During an EEG‐NF session, participants are usually provided with a visual stimulus continuously modified as feedback of their EEG activity within multiple trials. Yet, there are no common standards across studies for the frequency at which this stimulus is modified from real‐time EEG changes. Here, we thus investigated whether the EEG spectral power of the classically trained frequency bands was influenced by: (i) the presentation of the same visual stimulus over multiple trials, (ii) the continuous modification of this stimulus, and (iii) the frequency at which this stimulus is modified over time. To the best of our knowledge, this study is the first to properly investigate the natural time course of classically trained frequency bands in EEG‐NF studies, and the impact of observed discrepancies in protocol design on these frequency bands.

## 2 Materials and Methods

### 2.1 Participants

Thirty‐two healthy young adult volunteers (*M*_age_ = 23.67 years, SD = 3.41, age range = 18–33; 23 females; 29 right‐handed [self‐reported]) were recruited in September 2023. All participants reported normal or corrected‐to‐normal vision, and no neurological and/or psychiatric disorders. Participants were enrolled via a learning platform at Aix‐Marseille University or through posts on the lab's channels. Student participants (*n* = 13) received course credits as compensation for participation. Participants in the pilot study (described in the Supporting Information) were different from those in the present study.

All participants gave written and informed consent in accordance with the Declaration of Helsinki. Confidentiality was preserved, and an anonymous code was assigned to each participant. The experimental procedure was approved by the French Personal Protection Committee (CPP Sud Méditerranée V, ref. 19.09.12.44636).

### 2.2 Material

To mimic the time structure of a single EEG‐NF session, the task was composed of four conditions of eight 60‐s trials, each trial corresponding to the presentation of a gray circle at the centre of a blank screen. In the first, control condition, the size of the circle remained constant across trials (radius 100 pixels). In the remaining three conditions, the circle size was continuously modified during the trials. The continuous modification was performed at three different frequencies, respectively corresponding to the three remaining conditions: 1, 5, or 10 Hz. These frequencies were chosen to reproduce common frequencies at which feedback is updated in EEG‐NF protocols in healthy adults (Berger and Davelaar 2018; Boe et al. 2014; Enriquez‐Geppert, Huster, Figge, and Herrmann 2014; Enriquez‐Geppert, Huster, Scharfenort, et al. 2014; Hsueh et al. 2016; Kober, Witte, et al. 2015; Salari et al. 2014; Studer et al. 2014; Wei et al. 2017). Except for the characteristics of circle size modification (range of size variation at each time point, modification frequency), all characteristics of the circle remained constant across trials and conditions (color, maximum and minimum size).

A partial Latin‐square design was used to counterbalance the order of conditions across subjects. An in‐house Matlab script randomly generated four quadruplets (2143, 1432, 3214 and 4321) to ensure each condition appeared in every temporal position. Each participant was then pseudo‐randomly assigned one quadruplet, resulting in an even distribution.

### 2.3 Visual Stimulus Modification

During each trial, a gray circle was presented on the centre of a blank screen. In the control condition, the circle remained the same across trials. Within the other three conditions, the circle size changed at different frequency rates depending on the condition: 1, 5 or 10 Hz (i.e., changed every 1000, 200, or 100 ms, respectively). Possible values for the circle size changes were determined prior to data collection using EEG data from a previous pilot study. At each time point of a trial, the change in circle size was defined by randomly selecting one from these possible values. In EEG‐NF protocols, the feedback stimulus is continuously modified based on the real‐time fluctuations of an EEG spectral feature. These fluctuations follow a specific oscillatory pattern over time, reflecting the activity of neuron populations (Cohen 2017). By basing the circle size changes on dynamics observed in previously recorded EEG activity, we sought to present time‐point changes that visually mimicked those observed during a genuine EEG‐NF session. The pilot study was similar to the control condition of the present experiment in design, EEG recording, apparatus and population. We used the variations in the alpha band (8–12 Hz) spectral power during this pilot to generate the variations of the circle in the present study. Details about the pilot study and the procedure to generate current circle sizes can be found in the Supporting Information. Individual distributions of alpha power variations across pilot participants and corresponding derived circle size variations are shown in Figure S1. Figure S2 illustrates that the temporal structure of the circle size variations as implemented in our study is visually similar to the temporal structure of those generated from pilot data, that is, from actual variations of alpha spectral power.

### 2.4 Apparatus

The task and simultaneous EEG data acquisition were implemented in Matlab Release 2023a (Mathworks Inc.) using a DELL Mobile 3571 computer running Ubuntu 22.04 OS, and a NVIDIA T600 Laptop GPU. Specifically, EEG data acquisition required the Brainflow library version 5‐8‐1 and was done using an OpenBCI Cyton 8‐channels board, with OpenBCI Gold cup and Earclip electrodes. The visualization task was implemented using Psychtoolbox‐3 (Kleiner et al. 2007), and was displayed on a flat‐screen computer monitor (DELL P2419H) with a screen resolution of 1920 × 1080 pixels at a refresh rate of 60 Hz. During the task, the distance between the monitor (screen size 52.704 × 29.646 cm) and the back of the chair was kept constant. The distance ranged from 90 – 100 cm depending on the participant. EEG data was processed in Matlab Release 2023a (Mathworks Inc.). Statistical analyses and figures were performed in R version 4.3.3 (R Core Team 2024).

### 2.5 EEG Recording

EEG data was digitalised at 250 Hz in microvolts (μV) from the OpenBCI board in Matlab R2023a matrices. Data acquisition was done using the laptop's GPU to minimize computation time. We recorded the EEG signal from six OpenBCI Gold Cup electrodes placed in accordance with the 10–20 International System at the following positions: Fp1, Fpz, Fp2, Fz, Cz, and Pz. Two OpenBCI earclip electrodes placed on the left and the right earlobes were used as a reference for all electrodes and as a noise‐canceling ground electrode, respectively. Impedance was kept below 10 kΩ.

### 2.6 Procedure

Participants were seated in front of a monitor throughout the experiment. After obtaining written and informed consent, the EEG setup was installed and impedance checked. Participants were submitted to a passive visualization task, while their EEG activity was recorded. To mimic the conditions of an EEG‐NF session, participants were repeatedly asked to look at the same visual patterns on the monitor. Before the start of the task, participants were given verbal instructions (in French) about the design of the task: “You will complete 4 blocks of 8 trials, each lasting one minute. During each trial, a circle will be presented in the centre of the screen. During the 8 trials of a block, the circle can either remain the same, or its size will be changed continuously at the same rate. Your only task is to keep your eyes on the circle. There are no other particular instructions.” To avoid disturbing the EEG signal, participants were also asked to remain as calm and relaxed as possible during the trials. To start a new trial, participants had to press the “Enter” key on a keyboard placed between them and the screen. All participants were then free to take self‐paced breaks between trials. During the task, the experimenter (J.M.) remained in the room, but out of the participant's field of vision.

### 2.7 EEG Processing

In accordance with the guidelines for spectral analyses proposed by Keil et al. (2022) (see Table S1 for the corresponding completed checklist), EEG data processing was performed using in‐house Matlab scripts and EEGLAB (Delorme and Makeig 2004) in order to compute spectral power of theta (4–8 Hz), alpha (8–12 Hz), SMR (12–15 Hz), and beta (15–30 Hz) frequency bands. Given that neurofeedback studies traditionally do not propose an explicit model for the generation of oscillatory activity and the 1/*f* noise (Enriquez‐Geppert et al. 2017), we adopted the narrowband model implicitly assumed in the field for analyses (Keil et al. 2022). As a first step, data from all channels was zero‐phase filtered using the Matlab *filtfilt* function with a 0.5 Hz high‐pass filter (6th order IIR Butterworth) and a 50 Hz notch filter (2nd order IIR). This function ensures that the phase relationships in the EEG signal were preserved, avoiding distortions introduced by group delay or non‐linear phase filtering. For each trial data, the first 2 s and the last one were removed to delete the filter transients, resulting in segments of 57 s. Filtered data was then imported in EEGLAB and an extended Infomax Independent Component Analysis (ICA) was applied (Delorme et al. 2007). ICA components for eye blinks and lateral eye movements were identified and subtracted from the data by visual inspection of the component scalp topography, time series, and power spectrum distributions. For each participant, one to three components were removed (exact numbers of components removed by participant are reported in Table S2). The resulting EEG filtered and artifact‐corrected data was re‐imported in Matlab format.

At this stage, only data from Fz, Cz, and Pz electrodes was kept for further analyses. We used the Matlab function *pspectrum* to analyze EEG signals in the frequency domain. This function computed power spectra using FFT and by default applies Welch's method to improve robustness of spectral estimates. Specifically, the function automatically divides the signal into the longest possible segments to achieve as close to (but not exceeding) 8 segments with 50% overlap. Each segment is windowed using a Hamming window before computing the FFT, and the resulting power spectra are averaged to provide the final spectral estimates. For our dataset, *pspectrum* applied a frequency resolution of ~0.305 Hz, corresponding to the segment length determined by the above criteria. Spectral power estimates were computed for each participant and trial, and subsequently transformed to decibels (dB) to favor normally distributed data.

Finally, we extracted the power estimates of each frequency in the ranges of 4–8, 8–12, 12–15, and 15–30 Hz. The power estimates of each frequency range were averaged to obtain spectral power of theta, alpha, SMR and beta frequency bands, respectively.

### 2.8 Statistical Analyses and Hypotheses Testing

The resulting 12 dependent variables (i.e., spectral power of theta, alpha, SMR and beta frequency bands measured by Fz, Cz, and Pz electrodes) were *z*‐score standardized across subjects and used for statistical analyses. For the purpose of this study, we arbitrary hypothesized the presence of each effect of interest on these variables, that is, that their spectral power is influenced by (i) trial repetition, (ii) the continuous modification of the circle, and (iii) the frequency at which the circle is modified.

We fitted several Bayesian linear multilevel models using the brms and rstan R packages (Bürkner 2017; Stan Development Team 2024). Bayesian analyses provide several advantages for statistical analyses over frequentist equivalents (see Schad et al. (2021) and Schad et al. (2023) for extended tutorials on the use of Bayesian analyses), including the robustness to low‐power situations (Schönbrodt and Wagenmakers 2018), the facilitation of developing and fitting multilevel (or hierarchical/mixed) models (Gelman et al. 2014), the possibility to distinguish sensitive from insensitive evidence for an absence of effect (i.e., the null hypothesis *H*_0_, Dienes and Mclatchie 2018), and the robustness to multiple comparison issues (Gelman et al. 2012). Each model considered one of the above mentioned dependent variables as continuous, and included the maximal varying effect structure to account for the individual variability of subjects (Barr et al. 2013). As constant effects, we included the Trial number continuous predictor (i.e., integers from 1 to 8) and the Condition categorical predictor (i.e., Control, 1, 5, and 10 Hz), as well as their interaction. For the Trial continuous predictor, we defined the first trial as a reference for comparison. For the Condition categorical predictor, we followed Schad et al. (2020) guidelines to determine a custom contrast matrix for hypotheses testing via the generalized inverse. Since the contrasts were not orthogonal, applying the generalized matrix inverse ensured the proper computation of contrast weights and facilitated interpretation aligned with the hypotheses. The contrast matrix, shown in Table 1, was obtained by: (i) formulating the hypotheses, (ii) extracting the weights to construct a hypothesis matrix (Table S3), and (iii) applying the generalized matrix inverse to the hypothesis. This contrast matrix was assigned to the Condition predictor of each model, with the control condition as reference for comparison. Here is the full model equation (*brms* package syntax):

$$\text{Power}\sim 1+\text{Trial}\times \text{Condition}+\left(1+\text{Trial}\times \text{Condition}\right|\text{Subject})$$

Because the effect size of a classic EEG‐NF session is still not clearly identified, if even considered (Micoulaud‐Franchi and Fovet 2018; Schabus 2017; Thibault and Raz 2017; Vollebregt et al. 2014; Witte et al. 2018), we placed regularizing priors of *N*(0, 1) on each parameter, constraining the models to plausible values and avoiding overfitting issues (Schad et al. 2021). This prior indicates, for example to the model fitted on Fz alpha standardized power (*M*_power_ = −3.62 dB, SD = 2.63), that the effect of trial repetition will most likely be close to zero, with either a positive or negative sign, and has a 95% probability of lying between −2 and 2 standard deviations (SD) of alpha power distribution (i.e., between −5.25 and 5.25).

**TABLE 1:** Custom‐coded contrast matrix assigned to the condition predictor of each model.

Condition labels	Intercept	Experimental vs. Control (1st contrast)	5 Hz vs. 1 Hz (2nd contrast)	10 Hz vs. 5 Hz (3rd contrast)
Control	1	‐3/4	0	0
1 Hz	1	1/4	−2/3	−1/3
5 Hz	1	1/4	1/3	−1/3
10 Hz	1	1/4	1/3	2/3

*Note:* Within the four conditions composing our task, we manipulate the presence (three experimental conditions) or absence (Control) of the continuous modification of the circle size. We also manipulate, depending on the experimental condition, the frequency rate of this continuous modification: 1, 5, or 10 Hz. To include both predictors (i.e., the continuous modification of the circle size, and the frequency at which the circle was modified) in our models and relate to our hypotheses testing, we applied the present custom contrast matrix to the categorical predictor “Condition.” This matrix was obtained by applying the generalized inverse to the Hypothesis matrix (Table S3) referring to our hypotheses. The “Exp vs. Control (1st contrast)” column refers to the hypothesis that there is a difference in spectral power when participants are presented with a continuously modified circle (Experimental conditions), relative to when the circle remains the same (Control). The “5 Hz vs. 1 Hz (2nd contrast)” column relates to the hypothesis that there is a difference in spectral power when participants are presented a circle modified at 5 Hz relative to 1 Hz. The “10 Hz vs. 5 Hz (3rd contrast)” column relates to the hypothesis that there is a difference in spectral power when participants are presented a circle modified at 10 Hz relative to 5 Hz.

For each effect of interest, we computed Bayes Factors (BFs) to quantify the strength of evidence for a hypothesis over another (Dienes and Mclatchie 2018). To ensure we used enough Markov chain Monte Carlo (MCMC) draws to estimate stable BFs, we performed all reported statistical analyses five times (Schad et al. 2023). For each effect of interest, we report the mean of the five obtained posterior distributions, along with the largest limits of the 95% credible interval (CrI). We also report the mean of the obtained BF_10_ quantifying evidence for the presence of an effect (alternative hypothesis) over its absence (null hypothesis). As recommended by Jeffrey (1939), we consider that a BF_10_ of above 3 indicates substantial evidence for the alternative over the null hypothesis, and that a BF_10_ of below ⅓ substantial evidence for the null over the alternative hypothesis. A BF_10_ between ⅓ and 3 indicates data insensitivity to distinguish null and alternative hypotheses (Dienes 2014). When an effect was confirmed (i.e., BF_10_ > 3), we also reported the BF_10+_ quantifying the amount of evidence for a positive‐directional (i.e., one‐sided) effect. Estimates (standardized units) and BFs from each of the models regarding the predictors of interest are presented in Table S4.

Finally, since BFs are sensitive to priors, we conducted a sensitivity analysis by computing BFs for a range of priors to determine how these affected our conclusions (Schad et al. 2021). The range of priors assumed a priori effect sizes progressively restricted, with a 95% probability of lying between −2 and 2 SD, to between −0.4 and 0.4 SD. For each set of priors, we again computed each model five times to ensure BFs stability for inference. The results of this analysis are reported in the Supporting Information.

## 3 Results

### 3.1 Alpha Power Increases Spontaneously When Looking at the Same Stimulus Over Time

**FIGURE 1:**
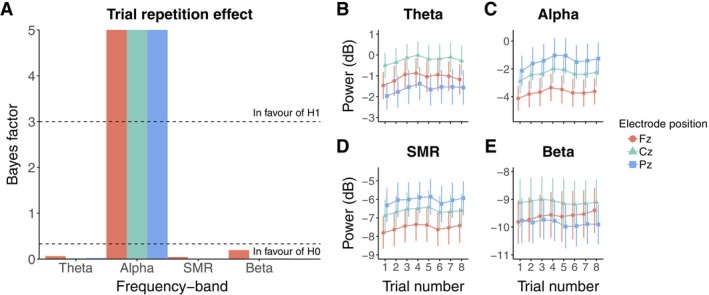
Evolution of EEG spectral power across the trials of the control condition. (A) BF10 quantifying evidence in favor of the alternative hypothesis (H1, i.e., the presence of an effect) over the null (*H*_0_, i.e., absence of effect) concerning the trial repetition effect on each of the frequency bands considered. The dashed horizontal line at y = 3 indicates the level of evidence above which the presence of an effect is favored (“In favour of H1”). The dashed horizontal line at *y* = ⅓ indicates the level of evidence under which the absence of an effect is favored (“In favour of H0”). (B–E) Respectively, evolution of theta (4–8 Hz), alpha (8–12 Hz), SMR (12–15 Hz) and beta (15–30 Hz) bands across the trials of the control condition. Each line point represents the EEG spectral power averaged at the group level. Error bars indicate 95% confidence intervals.

Figure 1 shows the evolution of the different frequency bands considered here (i.e., theta, alpha, SMR, beta) over the eight trials of the control condition, and the BF_10_ used to evaluate whether these frequency bands power remained stable across repeated trials. Figure 1A illustrates the observed moderate to strong evidence for a positive effect of trial repetition on the alpha spectral power at Fz (*β* = 0.02, 95% CrI [0.01, 0.029], BF_10_ = 7.642, BF_10+_ > 100), Cz (*β* = 0.023, 95% CrI [0.013, 0.032], BF_10_ = 17.402, BF_10+_ > 100) and Pz (*β* = 0.024, 95% CrI [0.013, 0.035], BF_10_ = 15.581, BF_10+_ > 100), indicating that the alpha power at Fz, Cz and Pz increases at each trial of the control condition (Figure 1C). For the other frequency bands of interest, we confirmed with moderate to extreme evidence the absence of an effect (Figure 1B,D,E). Model estimates regarding the trial repetition parameter are reported in Table 2.

**TABLE 2:** Estimates of trial repetition effect from models computed with hypothesized regularizing prior effect sizes of *N*(0, 1).

EEG band	Electrode	Estimate	Lower	Upper	BF_10_	BF_10+_
Theta	Fz	0.01	0.002	0.019	0.065	91.227
**Alpha**	**Fz**	**0.02**	**0.01**	**0.029**	**7.642**	**> 100**
SMR	Fz	0.01	0.001	0.019	0.045	56.728
Beta	Fz	0.018	0.004	0.032	0.196	> 100
Theta	Cz	0.008	−0.002	0.019	0.018	15.48
**Alpha**	**Cz**	**0.023**	**0.013**	**0.032**	**17.402**	> **100**
SMR	Cz	0.005	−0.003	0.014	0.009	8.295
Beta	Cz	0.001	−0.011	0.013	0.006	1.364
Theta	Pz	0.01	−0.003	0.023	0.023	16.617
**Alpha**	**Pz**	**0.024**	**0.013**	**0.035**	**15.581**	**> 100**
SMR	Pz	0.005	−0.003	0.013	0.008	7.397
Beta	Pz	−0.007	−0.021	0.007	0.012	0.174

*Note:* Each model reported has been computed five times to ensure the stability of the BFs. If not specified, each numerical value corresponds to the average of the values obtained across these five model computations. The “Estimate” column stands for the estimated group‐level effect (slope) of the “Trial” predictor considered in a model (in *z*‐score standardized units). The “Lower” and “Upper” columns correspond to the minimal lower and maximal upper bounds of the five 95% CrI computed. The “BF_10_” and “BF_10+_” columns correspond to the BF in favor of the alternative hypothesis (relative to the null) and the directional (i.e., one‐sided) BF, respectively. Rows in bold highlight the EEG features for which BFs quantify sufficient evidence in favor of the alternative hypothesis over the null (i.e., presence of an effect).

**FIGURE 2:**
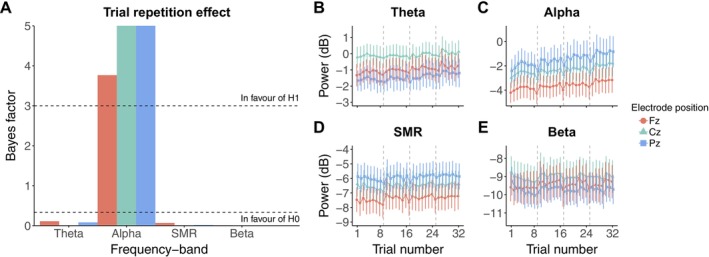
Evolution of EEG spectral power throughout the entire task. (A) BF_10_ quantifying evidence in favor of the alternative hypothesis (H1, i.e., the presence of an effect) over the null (*H*_0_, i.e., absence of effect) concerning the trial repetition effect over the entire task (regardless of experimental conditions) on each of the frequency bands considered. The horizontal dashed line at *y* = 3 indicates the level of evidence above which the presence of an effect is favored (“In favour of H1”). The horizontal dashed line at *y* = ⅓ indicates the level of evidence under which the absence of an effect is favored (“In favour of H0”). (B–E) Respectively, evolution of theta (4–8 Hz), alpha (8–12 Hz), SMR (12–15 Hz), and beta (15–30 Hz) bands across trials. Each line point represents the EEG spectral power averaged at the group level. Error bars indicate 95% confidence intervals. Vertical dashed lines mark the beginning of a condition (block of eight trials).

To assess in a longer run the natural fluctuations of alpha power, we exploratorily computed the same models by including only the Trial number throughout the entire experiment. To this end, we didn't consider the condition of trials (i.e., continuous modification of the circle size, frequency rate of the circle size modification). The continuous Trial predictor resulted in integers from 1 to 32. Figure 2 shows the evolution of the four frequency bands (i.e., theta, alpha, SMR and beta) over the 32 trials (independently of the experimental condition), and the BF_10_ used to estimate whether these frequency bands remain stable over the course of the task (Figure 2A). We also confirmed with moderate to extreme evidence that the alpha power at each electrode (Fz: *β* = 0.01, 95% CrI [0.005, 0.015], BF_10_ = 3.767, BF_10+_ > 100; Cz: *β* = 0.011, 95% CrI [0.006, 0.016], BF_10_ = 8.312, BF_10+_ > 100; Pz: *β* = 0.014, 95% CrI [0.009, 0.019], BF_10_ > 100, BF_10+_ > 100; see Figure 2C) increased throughout the entire task. Moderate to extreme evidence for no effect was observed for the power of theta, SMR, and beta frequency bands (Figure 2B,D,E). These results indicate that the alpha power at frontal (Fz), central (Cz) and posterior (Pz) electrodes spontaneously increases throughout the experiment. Model estimates are presented in Table S5.

### 3.2 Theta Power Is Influenced by the Perception of the Continuous Modification of the Visual Stimulus

**FIGURE 3:**
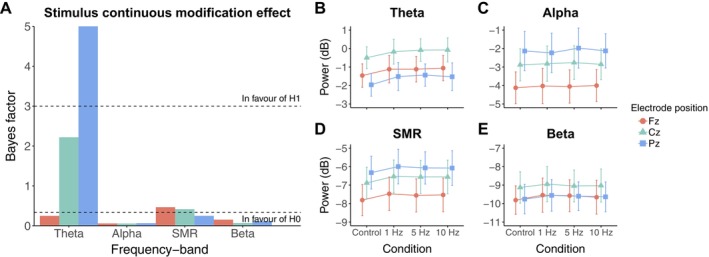
EEG spectral power at the first trial of each condition. (A) BF_10_ quantifying evidence in favor of the alternative hypothesis (H1, i.e., the presence of an effect) over the null (*H*_0_, i.e., absence of effect) concerning the effect of the continuous modification of the circle size on each of the frequency bands considered. The horizontal dashed line at *y* = 3 indicates the level of evidence above which the presence of an effect is favored (“In favour of H1”). The horizontal dashed line at *y* = ⅓ indicates the level of evidence under which the absence of an effect is favored (“In favour of H0”). (B–E) Respectively, theta (4–8 Hz), alpha (8–12 Hz), SMR (12–15 Hz) and beta (15–30 Hz) bands on the first trial of each condition. Each line point represents the EEG spectral power averaged at the group level. Error bars indicate 95% confidence intervals.

Figure 3 shows the BF_10_ used to estimate whether the frequency bands considered (i.e., theta, alpha, SMR and beta) are influenced by the continuous modification of the circle size (Figure 3A), and the spectral power of these frequency bands during the first trial of each condition (i.e., Control, 1 Hz, 5 Hz and 10 Hz; Figure 3B–E). We founded substantial evidence for an increase in theta power at Pz (*β* = 0.165, 95% CrI [0.065, 0.269], BF_10_ = 5.778, BF_10+_ > 100) when participants perceived a continuously modified circle compared to when the circle remained the same (Figure 3B). For the remaining frequency bands, moderate to extreme evidence for the absence of an effect was obtained (Figure 3C–E), except for the theta power at Cz (*β* = 0.149, 95% CrI [0.042, 0.255], BF_10_ = 2.219) and the SMR power at Fz (*β* = 0.103, 95% CrI [0.007, 0.198], BF_10_ = 0.465) and Cz (*β* = 0.096, 95% CrI [0.005, 0.187], BF_10_ = 0.416), where the BF_10_ was insensitive (i.e., between ⅓ and 3). Model estimates are presented in Table 3. Perceiving the continuous modification of the circle size thus induced an increase of theta power at Pz.

**TABLE 3:** Estimates of stimulus continuous modification effect from models computed with hypothesized regularizing prior effect sizes of *N*(0, 1).

EEG Band	Electrode	Estimate	Lower	Upper	BF_10_	BF_10+_
Theta	Fz	0.096	−0.08	0.219	0.247	20.424
Alpha	Fz	0.018	−0.091	0.127	0.057	1.701
SMR	Fz	0.103	0.007	0.198	0.465	55.859
Beta	Fz	0.087	−0.052	0.226	0.153	8.406
Theta	Cz	0.149	0.042	0.255	2.219	> 100
Alpha	Cz	−0.004	−0.119	0.11	0.057	0.89
SMR	Cz	0.096	0.005	0.187	0.416	52.248
Beta	Cz	0.029	−0.09	0.148	0.067	2.223
**Theta**	**Pz**	**0.165**	**0.062**	**0.269**	**5.778**	> **100**
Alpha	Pz	−0.015	−0.14	0.109	0.064	0.675
SMR	Pz	0.091	−0.012	0.194	0.249	24.208
Beta	Pz	0.069	−0.081	0.217	0.114	4.624

*Note:* Each model reported has been computed five times in order to ensure the stability of the BFs. If not specified, each numerical value corresponds to the average of the values obtained across these five model computations. The “Estimate” column stands for the estimated group‐level effect (slope) of the “Experimental conditions vs. control” predictor considered in a model (in *z*‐score standardized units). The “Lower” and “Upper” columns correspond to the minimal lower and maximal upper bounds of the five 95% CrI computed. The “BF_10_” and “BF_10+_” columns correspond to the BF in favor of the alternative hypothesis (relative to the null) and the directional (i.e., one‐sided) BF, respectively. Rows in bold highlight the EEG features for which BFs quantify sufficient evidence in favor of the alternative hypothesis over the null (i.e., presence of an effect).

Concerning the effect of the frequency of this continuous modification, Figure S3 shows that modifying the circle at 5 and 10 Hz, induces a peak in power spectra of the corresponding condition, whereas no such peak is observed at 1 Hz. However, the frequency of this continuous modification did not influence the power of all frequency bands and electrodes considered (see model estimates and BF_10_ for the comparisons in spectral power between the three modification frequencies in Table S4). The difference in power between the four conditions (control, modification at 1 Hz, modification at 5 Hz, modification at 10 Hz) is illustrated in Figure 3.

### 3.3 Absence of Interaction Between the Continuous Modification of the Circle (or Its Frequency Rate) and Trial Repetition

Finally, we founded strong to very strong support for an absence of interaction effects, that is, the interaction between trial repetition and: (i) the continuous modification of the circle size, and (ii) the frequency at which the circle was modified. These were evaluated in order to ensure that the evidence in favor of an effect for the Trial and Condition predictors is not restricted to the respective modality used as reference for subsequent comparisons (Trial 1 and the control condition, respectively). This suggests that the trial repetition effect on alpha power is independent of the continuous modification of the circle and its varying frequency rate, and that the effect of the continuous modification of the circle on theta power is observed no matter the trial number. The model estimates and BF_10_ corresponding to these interactions are reported in Table S4.

## 4 Discussion

In this study, healthy young adults participated to a passive visualization task mimicking the time structure and environment of an EEG‐NF session. During multiple trials, participants were asked to look at a gray circle which could either remain fixed (control condition) or be modified at three different frequency rates (experimental conditions 1, 5, and 10 Hz). Importantly, the present study demonstrates that: (i) the spectral power of the alpha (8–12 Hz) band increases spontaneously throughout the task, (ii) the spectral power of the theta (4–8 Hz) band is higher when perceiving the continuous modification of a visual stimulus (relative to perceiving the same fixed stimulus), and that (iii) none of the EEG frequency bands considered is influenced by the frequency at which the stimulus is modified. These results highlight the importance of considering confounding factors, inherent to EEG‐NF tasks, when assessing the efficacy of a protocol to modulate specific EEG features.

EEG‐NF protocols typically employ a closed‐loop system comprising at least four components: (i) EEG recording, (ii) extracting the targeted EEG feature(s) in real‐time, (iii) presenting a sensory feedback of these feature(s), and (iv) actively involving the participant in the self‐regulation of these feature(s) (Chiasson et al. 2023; Enriquez‐Geppert et al. 2017). During an EEG‐NF session, this closed‐loop system is implemented over multiple trials, with an usual session length of 20–40 min. As a result, a wide range of EEG spectral features (Chikhi et al. 2023; de Zambotti et al. 2012; Dekker et al. 2014; Enriquez‐Geppert, Huster, Scharfenort, et al. 2014; Eschmann et al. 2020; Escolano et al. 2014; Grosselin et al. 2021; Hoedlmoser et al. 2008; Janssen et al. 2017; Jurewicz et al. 2018; Kober et al. 2020; Li et al. 2023; Nan et al. 2015, 2020; Reis et al. 2016; Schabus et al. 2017; Singh et al. 2020; Zoefel et al. 2011) are modulated through an hypothesized self‐regulation mechanism (component iv of the closed‐loop system; Sitaram et al. 2017). Yet, to the best of our knowledge, this hypothesis still requires proper evaluation. Along with instructions for self‐regulation (component iv), the implementation of an EEG‐NF session involves setting up a common neuroscientific environment: EEG recording (components i and ii) concomitant to the presentation of a stimulus during repeated trials (component iii). At present, there is no evidence to suggest that this environment and its implementation over multiple trials do not already provide a sufficient framework to explain the usual EEG changes reported. Critically, the present findings provide evidence that EEG changes can occur in an EEG‐NF‐like task that lacks instructions for self‐regulation, challenging the accepted hypothesis of a single self‐regulation mechanism for EEG modulation. Overall, the role of each component of the EEG‐NF system currently needs to be properly evaluated.

The alpha band is one the most widely targeted frequency bands in EEG‐NF history (Micoulaud‐Franchi et al. 2021). In clinical settings, alpha power modulation is targeted in order to treat disorders identified with an alpha “abnormal” activity such as depressive (Choi et al. 2010; Linden 2014; Peeters et al. 2014) and anxiety (Hardt and Kamiya 1978; Plotkin and Rice 1981; Sandhu et al. 2007) disorders, post‐traumatic stress disorders (PTSD; Kluetsch et al. 2014; Peniston and Kulkosky 1991), alcohol addiction (Peniston and Kulkosky 1989; Saxby and Peniston 1995), headache (Andreychuk and Skriver 1975; Mathew et al. 1987; Stokes and Lappin 2010), and even chronical pain (Gannon and Sternbach 1971). Here, when passively looking at the same visual stimulus, we found that the participants alpha power at Fz, Cz, and Pz increases across the eight trials of a condition (Figure 1C). These results suggest that a spontaneous modulation of alpha power can occur without any actual feedback and without instructing participants to self‐modulate their EEG activity thanks to the latter.

Here, the alpha power does not only increase during a single condition of our task (i.e., over eight trials), but throughout the entire task, independently of the conditions (Figure 2C). Interestingly, an increase in alpha activity is often occurring during the different stages of EEG‐NF protocols (Chikhi et al. 2023; Naas et al. 2019; Rogala et al. 2016), even when alpha is not the trained frequency band (Dessy et al. 2020). More importantly, increases in alpha power with time is a documented issue in general experimental tasks unrelated to EEG‐NF (Benwell et al. 2019). Along with our results, this suggests that the observed alpha activity reflects processes that are not specifically engaged by EEG‐NF environment, independently of the hypothesized self‐regulating mechanism (Micoulaud Franchi et al. 2020). One possible explanation might lies in the boring nature of our task, boredom being closely linked to mind wandering, that is, focus attention on task‐unrelated thoughts (Blondé et al. 2022; Eastwood et al. 2012). As mind wandering has been shown to enhance alpha activity (Jin et al. 2019), it is very likely that participants concentrated progressively more on their thoughts throughout the task, which in turn exhibited an increase in alpha power.

During the present study, one can also visually notice that alpha power abruptly decreases at the first trial of each condition, before increasing again from the next one (Figure 2C). This is coherent with a previously observed “rebound” pattern occurring after a decrease in alpha EEG‐NF studies (Dempster and Vernon 2009; Zoefel et al. 2011; see in particular Figure 1 in Zoefel et al. 2011 which shows a very similar trend to ours). During the present task, participants were informed about the change in the visual patterns presented at each condition (i.e., “During the 8 trials of a block, the circle can either remain the same, or its size will be changed continuously at the same rate”). These abrupt negative deflections can therefore be interpretated as a shift in attentional focus towards the stimulus presented at the beginning of each condition. With trial repetition, as there was no more surprise in the visual patterns being presented, participants switched progressively to mind wandering. This hypothesis finds support in the latest framework presenting alpha activity as inhibited vs. exhibited by directed attention towards external (here the circle) versus internal (mind wandering) events, respectively (Cooper et al. 2003; Hanslmayr et al. 2011; Lou et al. 2014; Wang et al. 2016).

One could argue that these findings are not relevant for EEG‐NF. The present task is not a genuine EEG‐NF protocol, and then the results might not illustrate actual mechanisms engaged during EEG‐NF tasks. Yet, most of the models of EEG‐NF learning (i.e., how participants become able to self‐regulate their brain activity) consider internal events as an essential part for the self‐regulation to be achieved (Birbaumer et al. 2013; Davelaar 2018; Mirifar et al. 2022; Shibata et al. 2019; Wood et al. 2014). Some present the learning mechanism under the top‐down control of higher cognitive processes which, by trial and error, match the fluctuations of the external feedback to internal mental states (Bagdasaryan and Le Van Quyen 2013; Micoulaud‐Franchi et al. 2015). A core prediction of this theory would be that attentional focus fluctuates during EEG‐NF between external (feedback) and internal events (mental states). Aligned with our hypothesis about the top‐down directed attention mechanism of alpha activity, this would imply that fluctuations in alpha activity would be observed during EEG‐NF tasks. It is not surprising to notice that such alpha fluctuations are indeed reported (Chikhi et al. 2023; Dessy et al. 2020; Jiang et al. 2021; Naas et al. 2019). Hence, the present findings are consistent with EEG‐NF theories and previous empirical results, providing a solid framework to study the underlying mechanisms of EEG‐NF learning.

However, we remain aware that different interpretations exist (Birbaumer et al. 2013; Muñoz‐Moldes and Cleeremans 2020; Ros et al. 2014; Shibata et al. 2019), and that changes in alpha activity are not systematically reported during learning phases (e.g., Grosselin et al. 2021). Importantly, except for the absence of instructions to self‐regulate an EEG feature, the present task has strong similarities with sham EEG‐NF protocols. Sham protocols mimic genuine EEG‐NF ones, as participants are instructed to self‐regulate their brain activity based on the feedback fluctuations. However, without informing participants, the feedback is actually factice (not linked to the targeted EEG feature) and its fluctuations rely either on a different EEG feature, on a different individual's physiological signals, or on randomness. These protocols are used to control for non‐specific effects of EEG‐NF protocols on clinical or behavioral outcomes (Ros et al. 2020). Alternatively, the superiority of genuine over sham protocols is commonly observed in terms of intended EEG changes (Thibault and Raz 2018). Yet, by reviewing the studies employing a sham‐controlled procedure, we noticed that EEG changes can occur during sham protocols (Dessy et al. 2020; Jiang et al. 2021; Naas et al. 2019; Schabus et al. 2017). For instance, Schabus et al. (2017) evaluated, in patients with primary or misperception insomnia, the between‐session EEG changes in SMR amplitude in a genuine EEG‐NF compared to a sham. Their results indicate that SMR amplitude increases over both protocols, but that the increase is higher and quicker in the genuine protocol. Similarly, in healthy individuals, Naas et al. (2019) reported an increase of alpha amplitude during both sorts of protocols (no difference between the two), and Dessy et al. (2020) demonstrated within and between‐session changes in alpha and beta activity independently of their possibility to be trained. Thus, the present findings are consistent with the occurrence of EEG changes without providing participants with actual information about an EEG feature to modulate, nor the opportunity to modulate it. This highlights the need for systematic use of sham protocols to control for the specificity of the EEG changes targeted by EEG‐NF.

The EEG changes that occur during sham protocols, as in genuine EEG‐NF, may actually reflect a large variety of mechanisms. Sham protocols are designed to control for all non‐specific factors that might influence EEG‐NF outcomes (i.e., other than the self‐regulation mechanism, which cannot be engaged here because of the factice feedback). Usually, these non‐specific factors are considered to be either cognitive (engagement in self‐regulation, even if it is doomed to fail) and/or psychosocial (interaction with the practitioner, motivation, expectations, neurotechnological context; Thibault and Raz 2018). However, sham protocols also include non‐specific factors that are more general to the EEG‐NF procedure, such as time on task and the repeated exposure to the fluctuations of the same sensory feedback (Ros et al. 2020; Witte et al. 2018). Critically, the EEG changes reported during sham protocols (Dessy et al. 2020; Jiang et al. 2021; Naas et al. 2019; Schabus et al. 2017) could be due to one, several, or a combination of these factors. Furthermore, EEG changes reported in passive vs. active tasks, also in sham versus genuine EEG‐NF protocols, may elicit different underlying mechanisms. The present findings of a spontaneous increase in alpha power over time highlight the need to systematically assess the nature of the differences (if any) between sham and genuine protocols in terms of EEG changes. This issue is currently underestimated in the field, as the presence/source of EEG changes during sham protocols is overlooked, if even considered. For example, further research on alpha EEG‐NF might benefit from focusing on this issue by comparing the results of the present study with a sham EEG‐NF procedure. This would allow researchers to determine whether the increase in alpha power during sham is similar when there is no active cognitive and/or psychosocial engagement of participants in a self‐regulation task.

In this study, we also examined whether presenting a continuously modified stimulus, as opposed to an unmodified stimulus, influences the spectral power of EEG features commonly targeted by EEG‐NF. This question is crucial due to the lack of consensus on evidence‐based practices for presenting external feedback in EEG‐NF (Chiasson et al. 2023; Strehl 2014). A common approach is to provide positive feedback when the targeted EEG feature exceeds a threshold established during a “baseline” period, which is usually measured while the participant passively observes a fixation cross, similar to the control condition in this study (Agnoli et al. 2018; Dempster and Vernon 2009; Gonçalves et al. 2018; Maszczyk et al. 2020). However, using a baseline measure derived from passive observation poses significant issues. First, it assumes that the spectral features spontaneously fluctuate around their baseline, although recent evidence suggests this assumption should be critically examined (Benwell et al. 2019). Second, during genuine and sham EEG‐NF sessions, participants actively engage in a self‐regulation task, which can alter brain activity by engaging higher cognitive processes, potentially rendering the feedback threshold less effective as learning proxy (Micoulaud Franchi et al. 2020). Additionally, perceptual processes involved in observing a continuously modified stimulus can also affect the targeted EEG features, thus biasing the baseline measure. For example, the processing of visual stimuli influences either theta and/or alpha activity, which are often targeted in EEG‐NF (VanRullen 2016). Unsurprisingly, our findings demonstrated that perceiving a continuously modified stimulus, compared to an unmodified one, led to increased theta power at Pz. These results suggest that careful consideration is needed when using baselines to define feedback criteria, especially in protocols focusing on theta activity modulation at Pz (e.g., Egner and Gruzelier 2004; Rozengurt et al. 2016).

Beyond the threshold‐based feedback, another common practice involves continuously modifying a stimulus in proportion to the fluctuations of the targeted EEG feature (Berger and Davelaar 2018; Boe et al. 2014; Eschmann et al. 2022; Hsueh et al. 2016; Kober, Schweiger, et al. 2015; Salari et al. 2014). This method requires a predetermined frequency rate for stimulus modification, although standardization across the field is lacking. The last aim of this study was to assess whether variations in this modification frequency rate affect EEG activity. The data showed evidence for no impact on the spectral power of theta, alpha, SMR, and beta activities at Fz, Cz, and Pz. This absence of effect is reassuring, but the choice of feedback timing in EEG‐NF sessions should still not be based on arbitrary decisions, as participants do not engage in a self‐regulation task during the present study. Indeed, it remains unclear how such choices in feedback timing would impact self‐regulation performance during actual EEG‐NF session. The frequency of feedback presentation, whether at 1, 5, or 10 Hz, likely modifies the timing of information processing, potentially influencing the targeted EEG features (Fingelkurts and Fingelkurts 2006; Madl et al. 2011; Michel and Koenig 2018).

Given all results, the present study provides valuable insights for alpha and theta EEG‐NF protocols, particularly when assessing the targeted EEG modulation. At the same time, for many effects evaluated on many EEG features (theta power at Fz and Cz, SMR and beta power at Fz, Cz, and Pz), evidence for the absence of effect was consistently found. These findings are reassuring for the EEG‐NF field, as they suggest that most commonly targeted EEG features remain unaffected by the factors manipulated in the present study and inherent to EEG‐NF protocols (time on task, perception of a continuously modified stimulus, the frequency at which the stimulus is modified).

Moreover, several limitations must be acknowledged when interpreting these findings in relation to EEG‐NF. First, while the task shares similarities with EEG‐NF paradigms, it does not properly constitute a genuine or sham session. Such a session would require explicit participant engagement in self‐regulation, along with cognitive involvement, motivation, and expectations—all of which could potentially influence the observed EEG activity (Thibault and Raz 2017). It is plausible that the engagement of participants in a genuine or sham session could lead to alterations in the results owing to the presence of additional cognitive and psychosocial processes. Second and connectively, the continuous modification of the circle in this study was based on alpha power fluctuations measured at rest during a pilot study (see Supporting Information for details). While this approach aimed to replicate the natural time course of EEG variations at rest (see Figure S2), it may not fully replicate the EEG fluctuations observed in a genuine or sham EEG‐NF session, where additional processes (e.g., engagement, learning potential, motivation, expectations) likely play a role in shaping EEG activity. A final concern relates to the dynamics of the circle size variations. As indicated in the Method section, these variations were generated from the alpha variations in our pilot study, and Figure S1 shows the very good match between these variations. Nevertheless, post hoc analyses revealed that while skewness and kurtosis were similar in both studies, the overall variance and lag‐1 autocorrelations of circle size variations were dissimilar between studies, indicating discrepancies in the temporal dynamics of the circle size variations (see Figure S4). However, this limitation does not affect the main result of this study, that is, the increase in alpha power under all conditions and notably in the control condition where the circle size was fixed.

For too long, the field has focused on the establishment of EEG‐NF as an effective treatment for a wide range of disorders, attributing this efficacy to the intended EEG modulation (Thibault et al. 2018). Yet, in the light of the present study, we align with others claiming that characterizing the mechanism of action of EEG‐NF should be a prior step to its clinical application (Chiasson et al. 2023), especially when financial and ethical conflicts are steadily raised (Kalokairinou, Choi, et al. 2022; Nagappan et al. 2021). This study underscores the importance of further research to design optimal conditions to induce the EEG changes targeted through EEG‐NF, and to properly isolate them from non‐specific influences (Gruzelier 2014; Hasslinger et al. 2022).

In particular, we propose that future EEG‐NF studies systematically include two key control conditions. First, a sham EEG‐NF condition should be incorporated to differentiate the specific effect of self‐modulating a targeted EEG feature in a genuine session from the potential non‐specific effects observed in sham sessions, where participants believe they are engaging in self‐regulation but receive non‐contingent feedback. Second, an additional control condition similar to the present task should be used (i.e., a task in which the experimental conditions are the same as in an EEG‐NF sham condition but where there is no instruction to regulate brain activity but simply to pay attention to variations in visual information). Analyzing this condition alone provides insights into how confounding factors inherent to both sham and genuine EEG‐NF, such as time on task and the perception of continuously modified feedback, may influence the targeted EEG features. Furthermore, a comparison of this control condition with a sham session would allow researchers to isolate the contributions of cognitive and psychosocial factors, such as engagement, motivation, and expectations. Absent in a purely passive visualization task, these factors might influence the EEG changes targeted in EEG‐NF protocols. The implementation of these methodological improvements will enable future research to more rigorously dissociate specific EEG‐NF effects from general non‐specific influences on EEG activity (see Ninaus et al. 2013, for a similar approach in fMRI settings). Ultimately, this will deepen our understanding of EEG‐NF mechanisms and clarify the extent to which we can reasonably expect to induce specific EEG changes that drive (or not) substantial behavioral and clinical improvements.

## 5 Conclusion

Historically, the field of EEG‐NF has assumed that inducing specific EEG changes is a key prerequisite to expect clinical effectiveness (the so‐called “neurophysiological mechanisms”). Yet, recent evidence suggests that intended EEG changes are not responsible for the positive effects on clinical outcomes. Similarly, this study points out that the brain outcomes of EEG‐NF, that is, EEG spectral changes, can be induced by confounding factors inherent to EEG‐NF tasks (looking at the same visual patterns over iterative periods, looking at a continuously modified visual stimulus). It highlights that before claiming that EEG changes are (or are not) responsible for clinical improvements, one should be sure that the target EEG changes can be effectively induced by the hypothesized mechanisms. Further research along this probably long (and winding) road is therefore encouraged.

## Author Contributions

**Jacob Maaz:** conceptualization, data curation, formal analysis, investigation, methodology, resources, visualization, writing – original draft. **Véronique Paban:** conceptualization, methodology, project administration, resources, supervision, writing – review and editing. **Laurent Waroquier:** conceptualization, formal analysis, methodology, supervision, writing – review and editing. **Arnaud Rey:** conceptualization, methodology, project administration, resources, supervision, visualization, writing – review and editing.

## Disclosure

For the purpose of Open Access, a CC‐BY 4.0 public copyright license has been applied by the authors to the present document and will be applied to all subsequent versions up to the Author Accepted Manuscript arising from this submission.

## Conflicts of Interest

The authors declare no conflicts of interest.

## Supporting information


**Data S1:** psyp70163‐sup‐0001‐supinfo.docx.

## Data Availability

All materials, data, and analysis codes are available via the Open Science Framework at: https://osf.io/ep2kh/.
