# Supplementary material for: Spontaneous Modulation of Standard EEG Frequency Bands During a Neurofeedback‐Like Task
Source: Psychophysiology. 2025 Oct 4;62(10):e70163. doi: 10.1111/psyp.70163 (PMC12495449; doi:10.1111/psyp.70163)
Supplement: Supplementary file 1 — Data S1: psyp70163‐sup‐0001‐supinfo.docx. [file PSYP-62-e70163-s001.docx]

**Supplementary Material for:**

**Spontaneous modulation of standard EEG frequency bands during a neurofeedback-like task**

Jacob Maaz,^1,2,3^ Véronique Paban,^1^ Laurent Waroquier^4^ and Arnaud Rey^1,3^

Author affiliations:

1 Aix-Marseille Université, CNRS, CRPN, 13331 Marseille, France

2 Institute Neuro-Marseille, Aix-Marseille Université, France

3 Institute of Language Communication and the Brain, Aix-Marseille Université, France

4 Aix-Marseille Université, PSYCLE, 13621 Aix-en-Provence, France

Correspondence to: Jacob Maaz

Centre for Research in Psychology and Neuroscience (CRPN) – UMR 7077

CNRS – Aix-Marseille Université

3, place Victor Hugo – Case D

13331 Marseille Cedex 3 – France

[jacob.maaz@univ-amu.fr](mailto:jacob.maaz@univ-amu.fr)

# I – Method of the pilot study

## Participants

Ten healthy young adult volunteers (*M_age_* = 25.20 years, *SD* = 2.30, age range = 20-28; 7 females; 8 right-handed [self-reported]) were recruited. All participants reported normal or corrected-to-normal vision, and no neurological and/or psychiatric disorders. Participants were recruited through posts on the lab’s channels. All participants gave written and informed consent in accordance with the Declaration of Helsinki in June 2023. Confidentiality was preserved, and an anonymous code was assigned to each participant. The experimental procedure was approved by the French Personal Protection Committee (CPP Sud Méditerranée V, ref. 19.09.12.44636).

## Material

Participants were submitted to ten 90-second trials, each trial corresponding to the presentation of a fixed grey circle at the centre of a blank screen. All characteristics of the circle remained constant across trials.

## Apparatus

The task and simultaneous EEG data acquisition were implemented in Matlab Release 2023a (Mathworks, Inc.) using a DELL Mobile 3571 computer running Windows 10 OS. Specifically, EEG data was acquired using Lab Streaming Layer (LSL) and an OpenBCI [Cyton 8-channels board](https://docs.openbci.com/GettingStarted/Boards/CytonGS/), with Open BCI [Gold cup](https://shop.openbci.com/products/openbci-gold-cup-electrodes) and [Earclip](https://shop.openbci.com/products/earclip-electrode) electrodes. The visualisation task was displayed on a flat-screen computer monitor (DELL P2419H) with a screen resolution of 1920 × 1080 pixels at a refresh rate of 60 *Hz*. During the task, the distance between the monitor (screen size 52.704 × 29.646 cm) and the back of the chair was kept constant. EEG processing and subsequent time-frequency analyses was done in Matlab Release 2023a (Mathworks, Inc.).

## EEG recording

EEG data was digitalised at 250 *Hz* in microvolts (*µV*) from the OpenBCI board in Matlab R2023a matrices. We recorded the EEG signal from three OpenBCI Gold Cup electrodes placed in accordance with the 10-20 International System at the following positions: Fz, Cz, and Pz. Two OpenBCI earclip electrodes placed on the left and the right earlobes were used as a reference for all electrodes and as a noise-cancelling ground electrode, respectively. Impedance was kept below 10 *kΩ*.

## Procedure

Participants were seated in front of a monitor throughout the experiment. After obtaining written and informed consent, the EEG setup was installed and impedance checked. Participants were submitted to a passive visualisation task, while their EEG activity was recorded. Before the start of the task, participants were given verbal instructions (in French) about the design of the task: “You will complete 10 trials, each lasting one minute and a half. During each trial, a circle will be presented in the centre of the screen. Your only task is to keep your eyes on the circle. There are no other particular instructions.” To avoid disturbing the EEG signal, participants were also asked to remain as calm and relaxed as possible during the trials. Between trials, participants were free to take self-paced breaks. The experimenter manually ran a script to start each trial.

## EEG processing

EEG data processing was performed using in-house Matlab scripts. First, data from all channels was zero-phase filtered using a 0.5 *Hz* high-pass filter (6^th^ order IIR Butterworth) and a 50 *Hz* notch filter (2^nd^ order IIR). For each trial data, the first 2 seconds and the last one were removed to delete the filter transients, resulting in segments of 87 seconds. Three time-frequency analysis (moving-window short-FFT) was performed on each trial, electrode, and subject filtered data. A symmetric Hann window of 500 samples (2-second length) was used. At each step of the analyses, the spectral power estimates were computed in *dB*. The alpha band spectral power was then obtained by averaging power estimates of frequencies between 8 and 12 *Hz*.

Each of the three time-frequency analyses had an individual overlap between two consecutive windows. This was done to match the frequency at which the circle size was modified in the current experiment (i.e., 1, 5, or 10 *Hz*). For each analysis, the overlap was determined by dividing the EEG frequency sample by the corresponding circle modification frequency and subtracting the result from the window sample size. For example, for the 10 *Hz* condition, ten power values per second are required for the change in circle size to be determined by the change in alpha power. This resulted in a window overlap of: (500 - 250/10), or 475 samples. By keeping the same window size for all analyses, the frequency resolution was always 0.5 *Hz*. The averaged within-trial alpha power is presented in Supplementary Fig. 5.

## Simulations of circle sizes

For each participant of the pilot study, we simulated what the circle size would be (in each frequency case) if it depended linearly on alpha power during a trial. To simulate circle sizes linearly dependent on individual alpha power, we first resolved, for each pilot participant, an equation with two unknown factors *a* and *b*:

$$c =a \times p+b$$

with *c* referring to the circle size in pixels and *p* to the alpha power in dB.

To resolve the equation, we fixed a maximal and a minimal limit to circle size as 300 and 50 pixels, respectively, and considered the individual maximal and minimal alpha power computed for each subject. At each step of the time-frequency analysis, we used Equation 1 to simulate the corresponding individual circle size. We further calculated the difference between each two consecutive circle sizes generated.

This procedure was applied separately to the output of each three time-frequency analyses. To obtain finer distributions, we then brought together the values calculated from each subject’s individual data. As a result, we obtained three distributions of 25800, 127800 and 255300 possible circle sizes (accordingly, for a modification frequency of 1, 5 and 10 Hz), and, respectively, three distributions of 25500, 127500 and 255300 possible circle size variations (i.e., differences between two successive generated circle sizes). Each of these pairs of distributions was respectively matched to one experimental condition of the present study.

## Code and data accessibility

Materials, data, and analyses codes are available via the Open Science Framework at: <https://osf.io/wv2ta/>

# II – Results of the sensitivity analysis

## Trial repetition effect on alpha power

The sensitivity analysis suggested there was stronger evidence for *a priori* hypothesised smaller sizes (relative to the initial regularising prior of *N*(0, 1)) of the trial repetition effect on alpha power (Supplementary Fig. 6A). These results are coherent with the size of the estimated group-level effect at each electrode (Fz: *β* = 0.02, 95% CrI [0.01, 0.029]; Cz: *β* = 0.023, 95% CrI [0.013, 0.032]; Pz: *β* = 0.024, 95% CrI [0.013, 0.035]) which has a more restricted distribution than the one initially hypothesised. Model estimates and corresponding BFs of the sensitivity analysis are presented in Supplementary Table 6.

## Effect of the continuous modification of the circle size on theta power

As for the trial repetition effect on alpha power, the sensitivity analysis suggested that *a priori* hypothesised smaller sizes for the continuous modification effect on theta power at Pz are more plausible (Supplementary Fig. 6B). Concerning the theta power at Cz, inconclusive evidence (*BF_10_* between ⅓ and 3) became moderately in favour of an effect when reducing the hypothesised effect size with prior SDs of 0.6 (*β* = 0.148, 95% CrI [0.047, 0.255], *BF_10_* = 3.409, *BF_10+_* > 100), 0.4 (*β* = 0.146, 95% CrI [0.041, 0.252], *BF_10_* = 5.1, *BF_10+_* > 100) and 0.2 (*β* = 0.139, 95% CrI [0.036, 0.241], *BF_10_* = 7.853, *BF_10+_* > 100). Regarding theta power at Fz, evidence for the absence of an effect turned into inconclusive evidence with reduced prior SD 0.8 (*β* = 0.103, 95% CrI [-0.013, 0.219], *BF_10_* = 0.356), 0.6 (*β* = 0.103, 95% CrI [-0.014, 0.219], *BF_10_* = 0.468), 0.4 (*β* = 0.101, 95% CrI [-0.014, 0.216], *BF_10_* = 0.679) and 0.2 (*β* = 0.096, 95% CrI [-0.015, 0.206], *BF_10_* = 1.227). Overall, this highlights the robustness of the effect on Cz and Pz with more realistic hypothesised sizes, and the importance of considering prior assumptions for Bayesian inference. Supplementary Table 7 present the model estimates and BFs regarding this sensitivity analysis.

# III – Supplementary files

**Supplementary Table 1 Checklist guidelines for spectral analyses (adapted from Keil et al., 2022).**

| **#** | **Information to be included in the manuscript** | **Completed?** |
| --- | --- | --- |
| 1 | Specifying the inputs and outputs of all algorithms used in the processing pipeline | YES |
| 2 | A discussion of how oscillatory activity was conceptualised relative to 1/*f* noise and/or other broadband phenomena (underlying model) | YES |
| 3 | A rationale for the choice of measurement of power in a specific frequency band, including how nonperiodic (1/*f*) contributions to the spectrum were addressed | YES |
| 4 | A statement describing the specific type of Fourier- or non-Fourier-based algorithm used for transformation from the time domain to the frequency domain | YES |
| 5 | The exact duration of time segment used for transformation into the frequency domain for each condition of interest. In addition, the total number of segments (e.g., trials per condition) entering an averaged spectrum, along with how data epochs were combined within and across recordings (e.g., overlapping windows) | YES |
| 6 | The type, total number of, overlap between, and duration of any taper window functions, along with their ramp-on and ramp-off duration. If alternative and/or additional steps were taken to address edge artifacts, these should be stated. If applicable, the choice of taper window function should be specified as being guided by computational principles and/or by aiming to replicate current methods (e.g., Hann or Hamming window) | YES |
| 7 | If zero-padding is applied, the number and location of added zeros (e.g., before the time series, after the time series, or both before and after the time series) | Not applicable |
| 8 | All normalisation steps (e.g., by length of time, multiplication of the lower half of the spectrum, or by complex conjugate, etc.) applied to the spectral power or power density calculation | YES |
| 9 | The native frequency resolution of the spectrum (e.g., 1/(epoch duration in seconds)). In addition, the number of frequency bins extracted for a specific band of interest, and the range of these binds (e.g., 7.98 Hz to 11.97 Hz) | YES |
| 10 | Whether analyses were conducted using single trials or the average across trials | YES |
| 11 | How band power was measured from a spectrum | YES |

**Supplementary Table 2 Number of Independent Components removed from the data of each participant.**

| **Participant’s number** | **Number of Independent Components removed** |
| --- | --- |
| 1 | 2 |
| 2 | 2 |
| 3 | 2 |
| 4 | 2 |
| 5 | 2 |
| 6 | 2 |
| 7 | 2 |
| 8 | 2 |
| 9 | 2 |
| 10 | 2 |
| 11 | 2 |
| 12 | 2 |
| 13 | 2 |
| 14 | 3 |
| 15 | 3 |
| 16 | 2 |
| 17 | 2 |
| 18 | 2 |
| 19 | 1 |
| 20 | 2 |
| 21 | 2 |
| 22 | 2 |
| 23 | 2 |
| 24 | 3 |
| 25 | 2 |
| 26 | 1 |
| 27 | 3 |
| 28 | 2 |
| 29 | 2 |
| 30 | 1 |
| 31 | 2 |
| 32 | 2 |

All components were identified using the EEGLAB extended Infomax Independent Component Analysis (ICA) algorithm (Delorme et al., 2007). Independent Components for eye blinks and lateral eye movements were identified for rejection and subtracted from the data by visual inspection of the component scalp topography, time series, and power spectrum distributions. Note that, unusually, three components were removed from the data of four participants (participants 14, 15, 24 and 27). This was done because the ICA algorithm split one of the two typical eye artifact components (one for eye blink and one for lateral eye movements) into two different components. For example, ICA on participant 14’s data resulted in a duplication of the typical eye blink component. However, as removing more than two components in our setup (limited to six electrodes) might have compromised data integrity, we re-analysed the EEG data by limiting the number of components removed to two. The same reported statistical analyses were performed on these data. As they lead to the same conclusions as those reported in this paper, the results are presented in Supplementary Table 8.

**Supplementary Table 3 *Hypothesis* matrix regarding the custom contrast coding of the categorical predictor of Condition (i.e., Control, 1 *Hz*, 5 *Hz*, and 10 *Hz*).**

| **Condition labels** | **Intercept** | **Exp *vs*. Control (1^st^ contrast)** | **5 *Hz* *vs*. 1 *Hz* (2^nd^ contrast)** | **10 *Hz* *vs*. 5 *Hz* (3^rd^ contrast)** |
| --- | --- | --- | --- | --- |
| Control | 1/4 | -1 | 0 | 0 |
| 1 *Hz* | 1/4 | 1/3 | -1 | 0 |
| 5 *Hz* | 1/4 | 1/3 | 1 | -1 |
| 10 *Hz* | 1/4 | 1/3 | 0 | 1 |

Within the four conditions composing our task, we manipulate the presence (three Experimental conditions) or absence (Control) of the continuous modification of the circle size. We also manipulate, depending on the experimental condition, the frequency rate of this continuous modification: 1 *Hz*, 5 *Hz*, or 10 *Hz*. To include both predictors (i.e., the continuous modification of the circle size, and the frequency at which the circle was modified) in our models and relate to our hypotheses testing, we applied a custom contrast matrix to the categorical predictor ‘Condition’. The custom contrast matrix (Table 1) was obtained by applying the generalised inverse to the current *Hypothesis* matrix. The ‘Exp *vs*. Control (1^st^ contrast)’ column refers to the hypothesis that there is a difference in spectral power when participants are presented with a continuously modified circle (Experimental conditions), relative to when the circle remains the same (Control). The ‘5 *Hz* *vs*. 1 *Hz* (2^nd^ contrast)’ column relates to the hypothesis that there is a difference in spectral power when participants are presented a circle modified at 5 *Hz* relative to 1 *Hz*. The ’10 *Hz* *vs*. 5 *Hz* (3^rd^ contrast)’ column relates to the hypothesis that there is a difference in spectral power when participants are presented a circle modified at 10 *Hz* relative to 5 *Hz*. Coefficients have been obtained by formulating the corresponding hypothesis of no-difference (null). For instance, the null hypothesis of the ‘Exp *vs*. Control (1^st^ contrast)’ column formulates that the difference in spectral power between the mean of the three experimental conditions (⅓µ_1_ *_Hz_* + ⅓µ_5_ *_Hz_* + ⅓µ_10_ *_Hz_*) and the control condition (-1µ_Control_) would be null.

**Supplementary Table 4 Estimates from models computed with hypothesised regularising prior effect sizes of *N*(0, 1).**

| **EEG Band** | **Electrode** | **Predictor** | **Estimate** | **Lower** | **Upper** | ***BF_10_*** | ***BF_10+_*** |
| --- | --- | --- | --- | --- | --- | --- | --- |
| Theta | Fz | Trial | 0,01 | 0,002 | 0,019 | 0.065 | 91.227 |
| Theta | Fz | Exp *vs*. Control | 0,096 | -0,08 | 0,219 | 0.247 | 20.424 |
| Theta | Fz | 5 *Hz* *vs*. 1 *Hz* | -0,01 | -0,142 | 0,109 | 0.059 | 0.782 |
| Theta | Fz | 10 *Hz* *vs*. 5 *Hz* | 0,054 | -0,088 | 0,266 | 0.104 | 4.288 |
| Theta | Fz | Trial:Exp *vs*. Control | -0,007 | -0,03 | 0,035 | 0.017 | 0.567 |
| Theta | Fz | Trial:5 *Hz* *vs*. 1 *Hz* | 0,003 | -0,041 | 0,033 | 0.017 | 1.862 |
| Theta | Fz | Trial:10 *Hz* *vs*. 5 *Hz* | -0,016 | -0,043 | 0,024 | 0.033 | 0.152 |
| **Alpha** | **Fz** | **Trial** | **0,02** | **0,01** | **0,029** | **7.642** | **> 100** |
| Alpha | Fz | Exp *vs*. Control | 0,018 | -0,091 | 0,127 | 0.057 | 1.701 |
| Alpha | Fz | 5 *Hz* *vs*. 1 *Hz* | -0,006 | -0,11 | 0,099 | 0.053 | 0.837 |
| Alpha | Fz | 10 *Hz* *vs*. 5 *Hz* | 0,014 | -0,122 | 0,149 | 0.069 | 1.379 |
| Alpha | Fz | Trial:Exp *vs*. Control | 0,005 | -0,015 | 0,025 | 0.011 | 2.261 |
| Alpha | Fz | Trial:5 *Hz* *vs*. 1 *Hz* | -0,009 | -0,032 | 0,014 | 0.016 | 0.27 |
| Alpha | Fz | Trial:10 *Hz* *vs*. 5 *Hz* | 0,005 | -0,019 | 0,029 | 0.013 | 1.977 |
| SMR | Fz | Trial | 0,01 | 0,001 | 0,019 | 0.045 | 56.728 |
| SMR | Fz | Exp *vs*. Control | 0,103 | 0,007 | 0,198 | 0.465 | 55.859 |
| SMR | Fz | 5 *Hz* *vs*. 1 *Hz* | -0,108 | -0,205 | -0,012 | 0.568 | 0.013 |
| SMR | Fz | 10 *Hz* *vs*. 5 *Hz* | 0,039 | -0,083 | 0,161 | 0.076 | 2.86 |
| SMR | Fz | Trial:Exp *vs*. Control | -0,005 | -0,025 | 0,015 | 0.011 | 0.464 |
| SMR | Fz | Trial:5 *Hz* *vs*. 1 *Hz* | 0,017 | -0,008 | 0,041 | 0.031 | 10.492 |
| SMR | Fz | Trial:10 *Hz* *vs*. 5 *Hz* | -0,004 | -0,028 | 0,02 | 0.013 | 0.577 |
| Beta | Fz | Trial | 0,018 | 0,004 | 0,032 | 0.196 | > 100 |
| Beta | Fz | Exp *vs*. Control | 0,087 | -0,052 | 0,226 | 0.153 | 8.406 |
| Beta | Fz | 5 *Hz* *vs*. 1 *Hz* | -0,053 | -0,224 | 0,118 | 0.103 | 0.361 |
| Beta | Fz | 10 *Hz* *vs*. 5 *Hz* | 0,079 | -0,095 | 0,254 | 0.132 | 4.53 |
| Beta | Fz | Trial:Exp *vs*. Control | -0,001 | -0,025 | 0,024 | 0.012 | 0.898 |
| Beta | Fz | Trial:5 *Hz* *vs*. 1 *Hz* | -0,001 | -0,038 | 0,036 | 0.018 | 0.915 |
| Beta | Fz | Trial:10 *Hz* *vs*. 5 *Hz* | 0,004 | -0,028 | 0,036 | 0.016 | 1.451 |
| Theta | Cz | Trial | 0,008 | -0,002 | 0,019 | 0.018 | 15.48 |
| Theta | Cz | Exp *vs*. Control | 0,149 | 0,042 | 0,255 | 2.219 | > 100 |
| Theta | Cz | 5 *Hz* *vs*. 1 *Hz* | 0,034 | -0,079 | 0,148 | 0.069 | 2.671 |
| Theta | Cz | 10 *Hz* *vs*. 5 *Hz* | 0,017 | -0,115 | 0,15 | 0.069 | 1.526 |
| Theta | Cz | Trial:Exp *vs*. Control | -0,011 | -0,034 | 0,012 | 0.018 | 0.208 |
| Theta | Cz | Trial:5 *Hz* *vs*. 1 *Hz* | 0,004 | -0,023 | 0,031 | 0.014 | 1.683 |
| Theta | Cz | Trial:10 *Hz* *vs*. 5 *Hz* | -0,019 | -0,045 | 0,006 | 0.04 | 0.072 |
| **Alpha** | **Cz** | **Trial** | **0,023** | **0,013** | **0,032** | **17.402** | **> 100** |
| Alpha | Cz | Exp *vs*. Control | -0,004 | -0,119 | 0,11 | 0.057 | 0.89 |
| Alpha | Cz | 5 *Hz* *vs*. 1 *Hz* | -0,01 | -0,12 | 0,1 | 0.056 | 0.744 |
| Alpha | Cz | 10 *Hz* *vs*. 5 *Hz* | -0,002 | -0,136 | 0,131 | 0.066 | 0.945 |
| Alpha | Cz | Trial:Exp *vs*. Control | 0,003 | -0,018 | 0,023 | 0.011 | 1.482 |
| Alpha | Cz | Trial:5 *Hz* *vs*. 1 *Hz* | -0,01 | -0,035 | 0,015 | 0.017 | 0.27 |
| Alpha | Cz | Trial:10 *Hz* *vs*. 5 *Hz* | 0,004 | -0,021 | 0,028 | 0.013 | 1.602 |
| SMR | Cz | Trial | 0,005 | -0,003 | 0,014 | 0.009 | 8.295 |
| SMR | Cz | Exp *vs*. Control | 0,096 | 0,005 | 0,187 | 0.416 | 52.248 |
| SMR | Cz | 5 *Hz* *vs*. 1 *Hz* | -0,078 | -0,171 | 0,015 | 0.182 | 0.053 |
| SMR | Cz | 10 *Hz* *vs*. 5 *Hz* | 0,054 | -0,054 | 0,163 | 0.09 | 5.338 |
| SMR | Cz | Trial:Exp *vs*. Control | -0,004 | -0,024 | 0,017 | 0.011 | 0.576 |
| SMR | Cz | Trial:5 *Hz* *vs*. 1 *Hz* | 0,012 | -0,013 | 0,037 | 0.02 | 4.86 |
| SMR | Cz | Trial:10 *Hz* *vs*. 5 *Hz* | -0,011 | -0,034 | 0,011 | 0.019 | 0.193 |
| Beta | Cz | Trial | 0,001 | -0,011 | 0,013 | 0.006 | 1.364 |
| Beta | Cz | Exp *vs*. Control | 0,029 | -0,09 | 0,148 | 0.067 | 2.223 |
| Beta | Cz | 5 *Hz* *vs*. 1 *Hz* | -0,066 | -0,166 | 0,034 | 0.117 | 0.108 |
| Beta | Cz | 10 *Hz* *vs*. 5 *Hz* | 0,114 | -0,024 | 0,251 | 0.269 | 18.593 |
| Beta | Cz | Trial:Exp *vs*. Control | 0,008 | -0,014 | 0,03 | 0.014 | 3.162 |
| Beta | Cz | Trial:5 *Hz* *vs*. 1 *Hz* | 0,001 | -0,026 | 0,027 | 0.013 | 1.093 |
| Beta | Cz | Trial:10 *Hz* *vs*. 5 *Hz* | -0,007 | -0,035 | 0,021 | 0.016 | 0.445 |
| Theta | Pz | Trial | 0,01 | -0,003 | 0,023 | 0.023 | 16.617 |
| **Theta** | **Pz** | **Exp *vs*. Control** | **0,165** | **0,062** | **0,269** | **5.778** | **> 100** |
| Theta | Pz | 5 *Hz* *vs*. 1 *Hz* | -0,004 | -0,134 | 0,126 | 0.065 | 0.9 |
| Theta | Pz | 10 *Hz* *vs*. 5 *Hz* | -0,035 | -0,179 | 0,109 | 0.08 | 0.455 |
| Theta | Pz | Trial:Exp *vs*. Control | -0,015 | -0,04 | 0,009 | 0.027 | 0.117 |
| Theta | Pz | Trial:5 *Hz* *vs*. 1 *Hz* | 0,006 | -0,022 | 0,035 | 0.016 | 2.007 |
| Theta | Pz | Trial:10 *Hz* *vs*. 5 *Hz* | -0,016 | -0,043 | 0,01 | 0.029 | 0.124 |
| **Alpha** | **Pz** | **Trial** | **0,024** | **0,013** | **0,035** | **15.581** | **> 100** |
| Alpha | Pz | Exp *vs*. Control | -0,015 | -0,14 | 0,109 | 0.064 | 0.675 |
| Alpha | Pz | 5 *Hz* *vs*. 1 *Hz* | -0,003 | -0,116 | 0,109 | 0.056 | 0.919 |
| Alpha | Pz | 10 *Hz* *vs*. 5 *Hz* | 0,021 | -0,122 | 0,164 | 0.075 | 1.594 |
| Alpha | Pz | Trial:Exp *vs*. Control | -0,003 | -0,026 | 0,02 | 0.012 | 0.656 |
| Alpha | Pz | Trial:5 *Hz* *vs*. 1 *Hz* | -0,014 | -0,04 | 0,012 | 0.024 | 0.156 |
| Alpha | Pz | Trial:10 *Hz* *vs*. 5 *Hz* | 0 | -0,024 | 0,024 | 0.012 | 1.031 |
| SMR | Pz | Trial | 0,005 | -0,003 | 0,013 | 0.008 | 7.397 |
| SMR | Pz | Exp *vs*. Control | 0,091 | -0,012 | 0,194 | 0.249 | 24.208 |
| SMR | Pz | 5 *Hz* *vs*. 1 *Hz* | -0,037 | -0,131 | 0,058 | 0.064 | 0.28 |
| SMR | Pz | 10 *Hz* *vs*. 5 *Hz* | 0,013 | -0,089 | 0,114 | 0.053 | 1.494 |
| SMR | Pz | Trial:Exp *vs*. Control | -0,006 | -0,027 | 0,014 | 0.012 | 0.36 |
| SMR | Pz | Trial:5 *Hz* *vs*. 1 *Hz* | -0,001 | -0,027 | 0,025 | 0.013 | 0.89 |
| SMR | Pz | Trial:10 *Hz* *vs*. 5 *Hz* | -0,002 | -0,024 | 0,019 | 0.011 | 0.7 |
| Beta | Pz | Trial | -0,007 | -0,021 | 0,007 | 0.012 | 0.174 |
| Beta | Pz | Exp *vs*. Control | 0,069 | -0,081 | 0,217 | 0.114 | 4.624 |
| Beta | Pz | 5 *Hz* *vs*. 1 *Hz* | -0,033 | -0,141 | 0,075 | 0.066 | 0.378 |
| Beta | Pz | 10 *Hz* *vs*. 5 *Hz* | 0,108 | -0,051 | 0,266 | 0.201 | 10.389 |
| Beta | Pz | Trial:Exp *vs*. Control | 0,007 | -0,023 | 0,036 | 0.016 | 2.071 |
| Beta | Pz | Trial:5 *Hz* *vs*. 1 *Hz* | -0,014 | -0,041 | 0,012 | 0.024 | 0.17 |
| Beta | Pz | Trial:10 *Hz* *vs*. 5 *Hz* | -0,008 | -0,039 | 0,024 | 0.018 | 0.468 |

Each model reported has been computed five times in order to ensure the stability of the BFs. If not specified, each numerical value corresponds to the average of the values obtained across these five model computations. The ‘Estimate’ column stands for the estimated group-level effect (slope) of each predictor considered in a model (in z-score standardised units). For the ‘Trial’ predictor, the estimate corresponds to the group-level effect of one trial of the control condition (modality of Condition predictor defined as reference for subsequent comparisons). For the ‘Exp vs. Control’, ‘5 *Hz* *vs*. 1 *Hz*’ and ‘10 *Hz* *vs*. 5 *Hz*’ predictors, the estimate refers to the group-level effect when comparing, during each condition first trial (modality of Trial predictor defined as reference for subsequent comparisons), the mean of the three experimental conditions (1 *Hz*, 5 *Hz*, 10 *Hz*) to the control condition, the 5 *Hz* Condition to the 1 *Hz* Condition, and the 10 *Hz* Condition to the 1 *Hz* Condition, respectively. The ‘Lower’ and ‘Upper’ columns correspond to the minimal lower and maximal upper bounds of the five 95% CrI computed. The ‘*BF_10_*’ and ‘*BF_10+_*’ columns correspond to the BF in favour of the alternative hypothesis (relative to the null) and the directional (i.e., one-sided) BF, respectively.

Lines in gold highlight the EEG features for which BFs quantify sufficient evidence in favour of the alternative hypothesis over the null (i.e., presence of an effect).

**Supplementary Table 5 Estimates from models considering only the ‘Trial’ predictor (integers of 1 to 32) throughout the entire task.**

| **EEG Band** | **Electrode** | **Estimate** | **Lower** | **Upper** | ***BF_10_*** | ***BF_10+_*** |
| --- | --- | --- | --- | --- | --- | --- |
| Theta | Fz | 0,007 | 0,002 | 0,013 | 0.111 | > 100 |
| **Alpha** | **Fz** | **0,01** | **0,005** | **0,015** | **3.767** | **> 100** |
| SMR | Fz | 0,005 | 0,001 | 0,008 | 0.068 | > 100 |
| Beta | Fz | 0,004 | -0,001 | 0,009 | 0.011 | 24.413 |
| Theta | Cz | 0,005 | -0,001 | 0,01 | 0.011 | 17.26 |
| **Alpha** | **Cz** | **0,011** | **0,006** | **0,016** | **8.312** | **> 100** |
| SMR | Cz | 0,003 | 0 | 0,006 | 0.012 | 40.159 |
| Beta | Cz | 0,001 | -0,003 | 0,006 | 0.003 | 2.662 |
| Theta | Pz | 0,008 | 0,002 | 0,014 | 0.083 | > 100 |
| **Alpha** | **Pz** | **0,014** | **0,009** | **0,019** | **> 100** | **> 100** |
| SMR | Pz | 0,004 | 0 | 0,007 | 0.019 | 64.179 |
| Beta | Pz | 0 | -0,005 | 0,004 | 0.002 | 0.836 |

Each model reported has been computed five times in order to ensure the stability of the BFs. If not specified, each numerical value corresponds to the average of the values obtained across these five model computations. The ‘Estimate’ column stands for the estimated group-level effect (slope) of the ‘Trial’ predictor considered in a model (in z-score standardised units). The ‘Lower’ and ‘Upper’ columns correspond to the minimal lower and maximal upper bounds of the five 95% CrI computed. The ‘*BF_10_*’ and ‘*BF_10+_*’ columns correspond to the BF in favour of the alternative hypothesis (relative to the null) and the directional (i.e., one-sided) BF, respectively.

Lines in gold highlight the EEG features for which BFs quantify sufficient evidence in favour of the alternative hypothesis over the null (i.e., presence of an effect).

**Supplementary Table 6 Estimates from models relating to the sensitivity analysis on the Bayes factors evaluating the effect of trial repetition on alpha power.**

| **Electrode** | **Estimate** | **Lower** | **Upper** | ***BF_10_*** | ***BF_10+_*** | **Prior SD** |
| --- | --- | --- | --- | --- | --- | --- |
| Fz | 0,02 | 0,01 | 0,029 | 29.65 | > 100 | 0,2 |
| Fz | 0,02 | 0,01 | 0,029 | 21.115 | > 100 | 0,4 |
| Fz | 0,02 | 0,01 | 0,029 | 10.347 | > 100 | 0,6 |
| Fz | 0,02 | 0,01 | 0,029 | 9.479 | > 100 | 0,8 |
| Fz | 0,02 | 0,01 | 0,029 | 7.642 | > 100 | 1 |
| Cz | 0,023 | 0,013 | 0,032 | > 100 | > 100 | 0,2 |
| Cz | 0,023 | 0,013 | 0,032 | 75.537 | > 100 | 0,4 |
| Cz | 0,023 | 0,013 | 0,032 | 94.317 | > 100 | 0,6 |
| Cz | 0,023 | 0,013 | 0,032 | > 100 | > 100 | 0,8 |
| Cz | 0,023 | 0,013 | 0,032 | 17.402 | > 100 | 1 |
| Pz | 0,024 | 0,013 | 0,035 | > 100 | > 100 | 0,2 |
| Pz | 0,024 | 0,013 | 0,035 | 45.127 | > 100 | 0,4 |
| Pz | 0,024 | 0,013 | 0,035 | 29.105 | > 100 | 0,6 |
| Pz | 0,024 | 0,013 | 0,035 | 18.07 | > 100 | 0,8 |
| Pz | 0,024 | 0,013 | 0,035 | 15.581 | > 100 | 1 |

Each model reported has been computed five times in order to ensure the stability of the BFs. If not specified, each numerical value corresponds to the average of the values obtained across these five model computations. The ‘Estimate’ column stands for the estimated group-level effect (slope) of the ‘Trial’ predictor considered in a model (in z-score standardised units). The ‘Lower’ and ‘Upper’ columns correspond to the minimal lower and maximal upper bounds of the five 95% CrI computed. The ‘*BF_10_*’ and ‘*BF_10+_*’ columns correspond to the BF in favour of the alternative hypothesis (relative to the null) and the directional (i.e., one-sided) BF, respectively. The ‘Prior SD’ column refers to the hypothesised standard deviation of the prior effect size distribution (mean of 0).

**Supplementary Table 7 Estimates from models relating to the sensitivity analysis on the Bayes factors evaluating the effect of circle continuous modification on theta power.**

| **Electrode** | **Estimate** | **Lower** | **Upper** | ***BF_10_*** | ***BF_10+_*** | **Prior SD** |
| --- | --- | --- | --- | --- | --- | --- |
| Fz | 0,096 | -0,015 | 0,206 | 1.227 | 21.69 | 0,2 |
| Fz | 0,101 | -0,014 | 0,216 | 0.679 | 23.403 | 0,4 |
| Fz | 0,103 | -0,014 | 0,219 | 0.468 | 24.079 | 0,6 |
| Fz | 0,103 | -0,013 | 0,219 | 0.356 | 24.589 | 0,8 |
| Fz | 0,096 | -0,08 | 0,219 | 0.247 | 20.424 | 1 |
| **Cz** | **0,139** | **0,036** | **0,241** | **8.477** | **> 100** | **0,2** |
| **Cz** | **0,146** | **0,041** | **0,252** | **5.1** | **> 100** | **0,4** |
| **Cz** | **0,148** | **0,041** | **0,255** | **3.409** | **> 100** | **0,6** |
| Cz | 0,148 | 0,042 | 0,255 | 2.747 | > 100 | 0,8 |
| Cz | 0,149 | 0,042 | 0,255 | 2.219 | > 100 | 1 |
| **Pz** | **0,155** | **0,054** | **0,255** | **20.275** | **> 100** | **0,2** |
| **Pz** | **0,163** | **0,06** | **0,265** | **13.466** | **> 100** | **0,4** |
| **Pz** | **0,165** | **0,061** | **0,268** | **9.338** | **> 100** | **0,6** |
| **Pz** | **0,165** | **0,061** | **0,268** | **7.263** | **> 100** | **0,8** |
| **Pz** | **0,165** | **0,062** | **0,269** | **5.778** | **> 100** | **1** |

Each model reported has been computed five times in order to ensure the stability of the BFs. If not specified, each numerical value corresponds to the average of the values obtained across these five model computations. The ‘Estimate’ column stands for the estimated group-level effect (slope) of the ‘Experimental conditions *vs*. control’ predictor considered in a model (in z-score standardised units). The ‘Lower’ and ‘Upper’ columns correspond to the minimal lower and maximal upper bounds of the five 95% CrI computed. The ‘*BF_10_*’ and ‘*BF_10+_*’ columns correspond to the BF in favour of the alternative hypothesis (relative to the null) and the directional (i.e., one-sided) BF, respectively. The ‘Prior SD’ column refers to the hypothesised standard deviation of the prior effect size distribution (mean of 0).

Lines in gold highlight the results for which BFs quantify sufficient evidence in favour of the alternative hypothesis over the null (i.e., presence of an effect).

**Supplementary Table 8 Estimates from models computed with hypothesised regularising prior effect sizes of *N*(0, 1) (data with max two Independent Components removed).**

| **EEG Band** | **Electrode** | **Predictor** | **Estimate** | **Lower** | **Upper** | ***BF_10_*** | ***BF_10+_*** |
| --- | --- | --- | --- | --- | --- | --- | --- |
| Theta | Fz | Trial | 0,01 | 0,001 | 0,019 | 0.053 | 76.047 |
| Theta | Fz | Exp *vs*. Control | 0,105 | -0,01 | 0,22 | 0.301 | 27.12 |
| Theta | Fz | 5 *Hz* *vs*. 1 *Hz* | -0,007 | -0,12 | 0,106 | 0.056 | 0.822 |
| Theta | Fz | 10 *Hz* *vs*. 5 *Hz* | 0,036 | -0,09 | 0,162 | 0.074 | 2.519 |
| Theta | Fz | Trial:Exp *vs*. Control | -0,011 | -0,031 | 0,01 | 0.017 | 0.18 |
| Theta | Fz | Trial:5 *Hz* *vs*. 1 *Hz* | 0,008 | -0,017 | 0,034 | 0.016 | 2.87 |
| Theta | Fz | Trial:10 *Hz* *vs*. 5 *Hz* | -0,018 | -0,042 | 0,007 | 0.036 | 0.08 |
| **Alpha** | **Fz** | **Trial** | **0,02** | **0,01** | **0,029** | **4.281** | **> 100** |
| Alpha | Fz | Exp *vs*. Control | 0,02 | -0,088 | 0,128 | 0.058 | 1.792 |
| Alpha | Fz | 5 *Hz* *vs*. 1 *Hz* | -0,005 | -0,108 | 0,1 | 0.052 | 0.87 |
| Alpha | Fz | 10 *Hz* *vs*. 5 *Hz* | 0,014 | -0,122 | 0,15 | 0.069 | 1.401 |
| Alpha | Fz | Trial:Exp *vs*. Control | 0,004 | -0,015 | 0,024 | 0.011 | 2.064 |
| Alpha | Fz | Trial:5 *Hz* *vs*. 1 *Hz* | -0,01 | -0,033 | 0,014 | 0.016 | 0.252 |
| Alpha | Fz | Trial:10 *Hz* *vs*. 5 *Hz* | 0,005 | -0,018 | 0,029 | 0.013 | 2.04 |
| SMR | Fz | Trial | 0,009 | 0,001 | 0,018 | 0.044 | 54.802 |
| SMR | Fz | Exp *vs*. Control | 0,107 | 0,013 | 0,202 | 0.571 | 75.147 |
| SMR | Fz | 5 *Hz* *vs*. 1 *Hz* | -0,108 | -0,203 | -0,013 | 0.546 | 0.014 |
| SMR | Fz | 10 *Hz* *vs*. 5 *Hz* | 0,042 | -0,08 | 0,164 | 0.078 | 3.082 |
| SMR | Fz | Trial:Exp *vs*. Control | -0,006 | -0,026 | 0,014 | 0.012 | 0.392 |
| SMR | Fz | Trial:5 *Hz* *vs*. 1 *Hz* | 0,017 | -0,008 | 0,041 | 0.03 | 10.322 |
| SMR | Fz | Trial:10 *Hz* *vs*. 5 *Hz* | -0,004 | -0,028 | 0,02 | 0.013 | 0.562 |
| Beta | Fz | Trial | 0,018 | 0,004 | 0,031 | 0.178 | > 100 |
| Beta | Fz | Exp *vs*. Control | 0,09 | -0,046 | 0,225 | 0.166 | 9.541 |
| Beta | Fz | 5 *Hz* *vs*. 1 *Hz* | -0,049 | -0,216 | 0,12 | 0.1 | 0.39 |
| Beta | Fz | 10 *Hz* *vs*. 5 *Hz* | 0,084 | -0,09 | 0,258 | 0.14 | 5.047 |
| Beta | Fz | Trial:Exp *vs*. Control | -0,001 | -0,025 | 0,023 | 0.012 | 0.879 |
| Beta | Fz | Trial:5 *Hz* *vs*. 1 *Hz* | -0,002 | -0,04 | 0,035 | 0.019 | 0.807 |
| Beta | Fz | Trial:10 *Hz* *vs*. 5 *Hz* | 0,004 | -0,028 | 0,035 | 0.016 | 1.478 |
| Theta | Cz | Trial | 0,008 | -0,002 | 0,019 | 0.017 | 14.777 |
| Theta | Cz | Exp *vs*. Control | 0,148 | 0,04 | 0,254 | 2.046 | > 100 |
| Theta | Cz | 5 *Hz* *vs*. 1 *Hz* | 0,033 | -0,08 | 0,146 | 0.068 | 2.59 |
| Theta | Cz | 10 *Hz* *vs*. 5 *Hz* | 0,016 | -0,114 | 0,148 | 0.068 | 1.49 |
| Theta | Cz | Trial:Exp *vs*. Control | -0,011 | -0,033 | 0,012 | 0.018 | 0.217 |
| Theta | Cz | Trial:5 *Hz* *vs*. 1 *Hz* | 0,005 | -0,022 | 0,032 | 0.015 | 1.744 |
| Theta | Cz | Trial:10 *Hz* *vs*. 5 *Hz* | -0,019 | -0,044 | 0,006 | 0.038 | 0.075 |
| **Alpha** | **Cz** | **Trial** | **0,023** | **0,013** | **0,032** | **> 100** | **> 100** |
| Alpha | Cz | Exp *vs*. Control | -0,004 | -0,118 | 0,11 | 0.056 | 0.885 |
| Alpha | Cz | 5 *Hz* *vs*. 1 *Hz* | -0,01 | -0,12 | 0,1 | 0.056 | 0.751 |
| Alpha | Cz | 10 *Hz* *vs*. 5 *Hz* | -0,003 | -0,135 | 0,13 | 0.066 | 0.933 |
| Alpha | Cz | Trial:Exp *vs*. Control | 0,002 | -0,018 | 0,023 | 0.011 | 1.473 |
| Alpha | Cz | Trial:5 *Hz* *vs*. 1 *Hz* | -0,01 | -0,035 | 0,015 | 0.017 | 0.263 |
| Alpha | Cz | Trial:10 *Hz* *vs*. 5 *Hz* | 0,004 | -0,021 | 0,028 | 0.013 | 1.647 |
| SMR | Cz | Trial | 0,005 | -0,003 | 0,014 | 0.009 | 8.177 |
| SMR | Cz | Exp *vs*. Control | 0,098 | 0,007 | 0,188 | 0.435 | 54.785 |
| SMR | Cz | 5 *Hz* *vs*. 1 *Hz* | -0,076 | -0,17 | 0,016 | 0.178 | 0.054 |
| SMR | Cz | 10 *Hz* *vs*. 5 *Hz* | 0,054 | -0,053 | 0,162 | 0.09 | 5.363 |
| SMR | Cz | Trial:Exp *vs*. Control | -0,004 | -0,024 | 0,016 | 0.011 | 0.544 |
| SMR | Cz | Trial:5 *Hz* *vs*. 1 *Hz* | 0,012 | -0,013 | 0,036 | 0.02 | 4.745 |
| SMR | Cz | Trial:10 *Hz* *vs*. 5 *Hz* | -0,011 | -0,034 | 0,011 | 0.018 | 0.192 |
| Beta | Cz | Trial | 0,001 | -0,01 | 0,013 | 0.006 | 1.386 |
| Beta | Cz | Exp *vs*. Control | 0,031 | -0,087 | 0,148 | 0.068 | 2.315 |
| Beta | Cz | 5 *Hz* *vs*. 1 *Hz* | -0,066 | -0,166 | 0,034 | 0.116 | 0.107 |
| Beta | Cz | 10 *Hz* *vs*. 5 *Hz* | 0,115 | -0,022 | 0,253 | 0.285 | 19.211 |
| Beta | Cz | Trial:Exp *vs*. Control | 0,008 | -0,014 | 0,029 | 0.014 | 3.121 |
| Beta | Cz | Trial:5 *Hz* *vs*. 1 *Hz* | 0,001 | -0,026 | 0,027 | 0.013 | 1.078 |
| Beta | Cz | Trial:10 *Hz* *vs*. 5 *Hz* | -0,007 | -0,035 | 0,021 | 0.016 | 0.443 |
| Theta | Pz | Trial | 0,01 | -0,003 | 0,023 | 0.024 | 16.69 |
| **Theta** | **Pz** | **Exp *vs*. Control** | **0,165** | **0,061** | **0,268** | **5.366** | **> 100** |
| Theta | Pz | 5 *Hz* *vs*. 1 *Hz* | 0,165 | 0,061 | 0,268 | 5.366 | > 100 |
| Theta | Pz | 10 *Hz* *vs*. 5 *Hz* | -0,005 | -0,134 | 0,124 | 0.066 | 0.889 |
| Theta | Pz | Trial:Exp *vs*. Control | -0,035 | -0,178 | 0,108 | 0.08 | 0.452 |
| Theta | Pz | Trial:5 *Hz* *vs*. 1 *Hz* | -0,015 | -0,04 | 0,009 | 0.027 | 0.117 |
| Theta | Pz | Trial:10 *Hz* *vs*. 5 *Hz* | 0,006 | -0,022 | 0,035 | 0.016 | 2.022 |
| **Alpha** | **Pz** | **Trial** | **0,024** | **0,013** | **0,035** | **11.641** | **> 100** |
| Alpha | Pz | Exp *vs*. Control | -0,015 | -0,139 | 0,11 | 0.063 | 0.679 |
| Alpha | Pz | 5 *Hz* *vs*. 1 *Hz* | -0,003 | -0,114 | 0,11 | 0.056 | 0.925 |
| Alpha | Pz | 10 *Hz* *vs*. 5 *Hz* | 0,02 | -0,122 | 0,164 | 0.074 | 1.575 |
| Alpha | Pz | Trial:Exp *vs*. Control | -0,003 | -0,026 | 0,02 | 0.012 | 0.663 |
| Alpha | Pz | Trial:5 *Hz* *vs*. 1 *Hz* | -0,014 | -0,04 | 0,011 | 0.024 | 0.156 |
| Alpha | Pz | Trial:10 *Hz* *vs*. 5 *Hz* | 0 | -0,024 | 0,024 | 0.012 | 1.055 |
| SMR | Pz | Trial | 0,005 | -0,003 | 0,013 | 0.008 | 7.302 |
| SMR | Pz | Exp *vs*. Control | 0,092 | -0,011 | 0,195 | 0.254 | 24.385 |
| SMR | Pz | 5 *Hz* *vs*. 1 *Hz* | -0,036 | -0,129 | 0,058 | 0.062 | 0.29 |
| SMR | Pz | 10 *Hz* *vs*. 5 *Hz* | 0,013 | -0,088 | 0,113 | 0.052 | 1.479 |
| SMR | Pz | Trial:Exp *vs*. Control | -0,007 | -0,027 | 0,014 | 0.012 | 0.354 |
| SMR | Pz | Trial:5 *Hz* *vs*. 1 *Hz* | -0,001 | -0,027 | 0,024 | 0.013 | 0.869 |
| SMR | Pz | Trial:10 *Hz* *vs*. 5 *Hz* | -0,002 | -0,024 | 0,019 | 0.011 | 0.703 |
| Beta | Pz | Trial | -0,007 | -0,021 | 0,007 | 0.012 | 0.18 |
| Beta | Pz | Exp *vs*. Control | 0,069 | -0,079 | 0,217 | 0.116 | 4.726 |
| Beta | Pz | 5 *Hz* *vs*. 1 *Hz* | -0,034 | -0,142 | 0,074 | 0.065 | 0.376 |
| Beta | Pz | 10 *Hz* *vs*. 5 *Hz* | 0,108 | -0,05 | 0,266 | 0.203 | 10.345 |
| Beta | Pz | Trial:Exp *vs*. Control | 0,006 | -0,022 | 0,036 | 0.016 | 2.067 |
| Beta | Pz | Trial:5 *Hz* *vs*. 1 *Hz* | -0,014 | -0,041 | 0,012 | 0.023 | 0.171 |
| Beta | Pz | Trial:10 *Hz* *vs*. 5 *Hz* | -0,008 | -0,04 | 0,024 | 0.018 | 0.474 |

Each model reported has been computed twice in order to ensure the stability of the BFs. If not specified, each numerical value corresponds to the average of the values obtained across these two model computations. The ‘Estimate’ column stands for the estimated group-level effect (slope) of each predictor considered in a model (in z-score standardised units). For the ‘Trial’ predictor, the estimate corresponds to the group-level effect of one trial of the control condition (modality of Condition predictor defined as reference for subsequent comparisons). For the ‘Exp vs. Control’, ‘5 *Hz* *vs*. 1 *Hz*’ and ‘10 *Hz* *vs*. 5 *Hz*’ predictors, the estimate refers to the group-level effect when comparing, during each condition first trial (modality of Trial predictor defined as reference for subsequent comparisons), the mean of the three experimental conditions (1 *Hz*, 5 *Hz*, 10 *Hz*) to the control condition, the 5 *Hz* Condition to the 1 *Hz* Condition, and the 10 *Hz* Condition to the 1 *Hz* Condition, respectively. The ‘Lower’ and ‘Upper’ columns correspond to the minimal lower and maximal upper bounds of the two 95% CrI computed. The ‘*BF_10_*’ and ‘*BF_10+_*’ columns correspond to the BF in favour of the alternative hypothesis (relative to the null) and the directional (i.e., one-sided) BF, respectively.

Lines in gold highlight the EEG features for which BFs quantify sufficient evidence in favour of the alternative hypothesis over the null (i.e., presence of an effect).


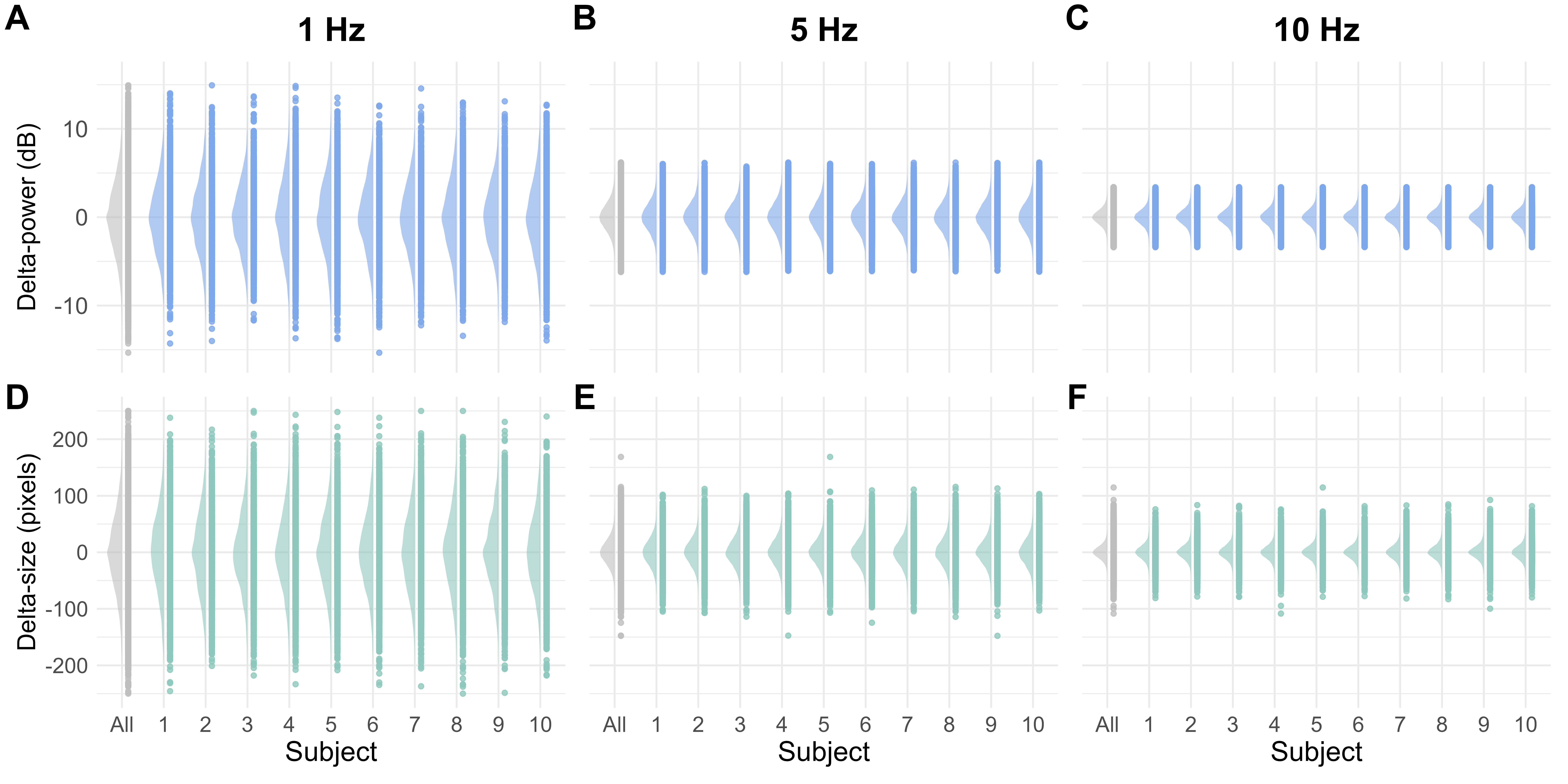
 **Supplementary Figure 1 Individual distribution of alpha power (8-12 Hz) variations and circle size variations of pilot participants.** (**A-C**) Variations of alpha power computed for each pilot participant at three different update frequencies during trials: 1 Hz (A), 5 Hz (B), and 10 Hz (C). Alpha power was computed using three time-frequency analyses with a 2-second Hann window and varying overlaps to match the update frequencies (50%, 90%, and 95%, respectively). On each plot, corresponding distributions of alpha variations for each participant are shown in blue, and the overall distribution in grey. (**D-F**) Respectively, simulated circle size variations derived from the alpha power variations in A-C, representing how circle size would vary if controlled by alpha power variations at the corresponding modification frequencies (i.e., 1 Hz in D, 5 Hz in E, and 10 Hz in F). On each plot, individual distributions for each pilot participant are shown in green, and in grey the aggregated data across all participants.

**
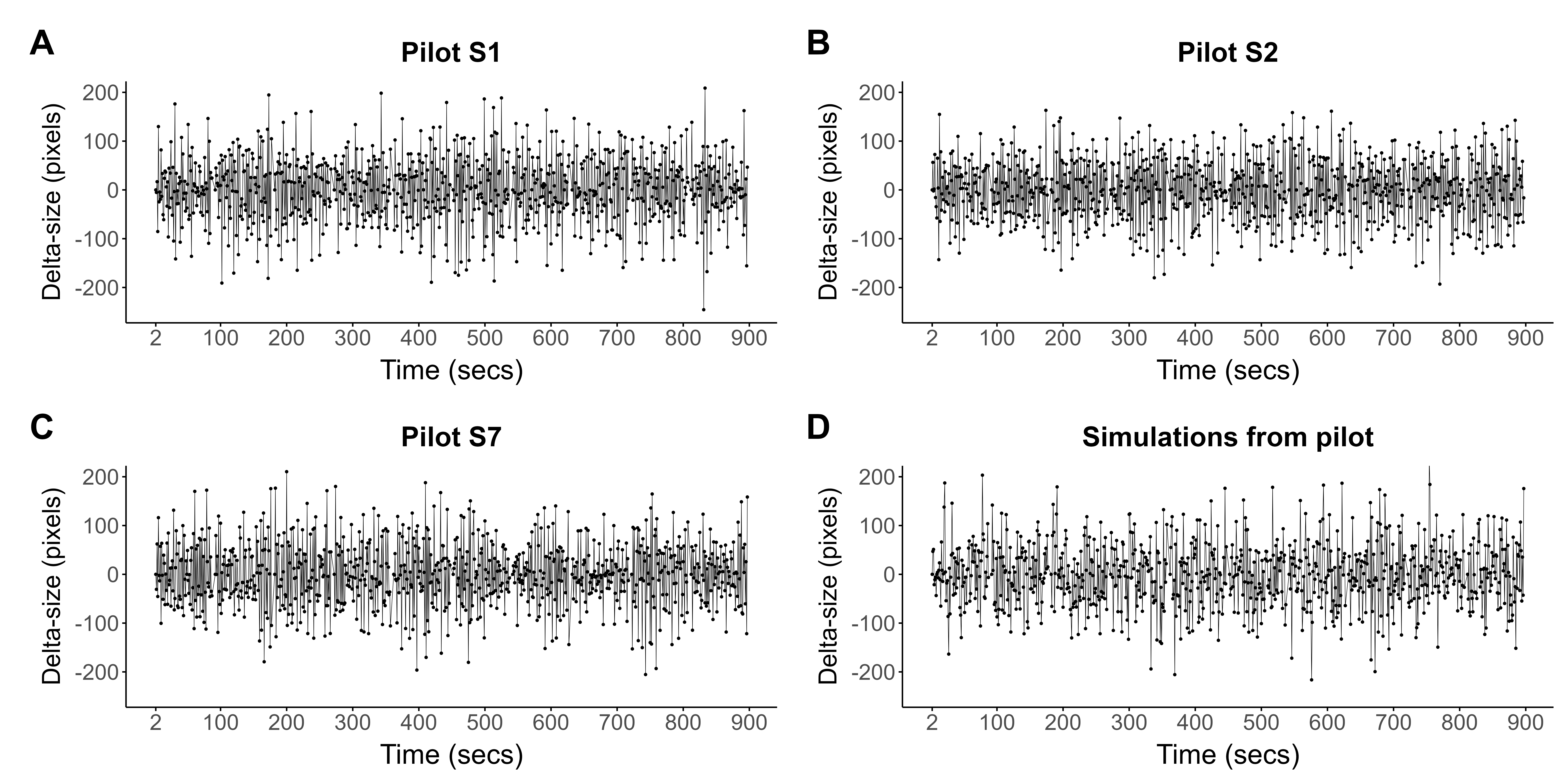
 Supplementary Figure 2 Circle size variations over time in the pilot study.** (**A-C**) Circle size variations generated for one pilot participant (i.e., S1, S2, S7, respectively) based on their alpha band (8-12 *Hz*) spectral power throughout the pilot experiment. Alpha power was computed using a time-frequency analysis on filtered data (symmetric Hann window of 500-sample length, 50% overlap, frequency resolution of ~0.305 *Hz*). For each participant, circle size was derived to visually represent alpha power fluctuations, as would occur during an actual neurofeedback session. Corresponding size variations were calculated as the difference between consecutive circle sizes. (**D**) Simulated circle size variations generated from the pilot data. As during the current study, each time point was randomly selected from the distribution of possible circle size variations generated from the pilot. Notably, the time course of simulated circle size variations (D) is visually difficult to distinguish from actual variations (A-C), suggesting sufficient fidelity of the simulation method to mimic neurofeedback time-point dynamics.

**
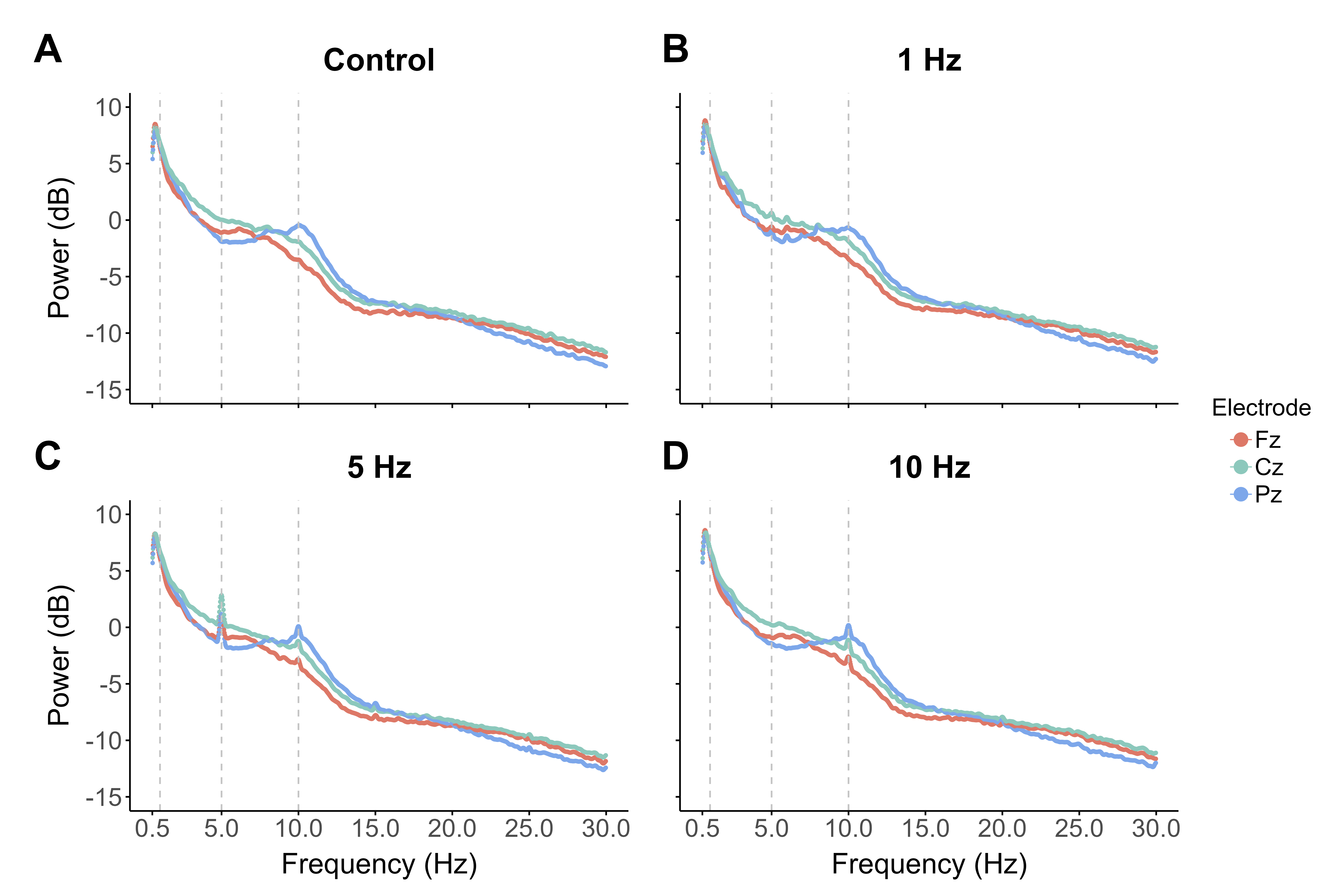
**

**Supplementary Figure 3 Averaged power spectra of each experimental condition.** Power spectra averaged across the group- and trial-levels of each experimental conditions: Control (**A**), 1 Hz (**B**), 5 Hz (**C**), and 10 Hz (**D**). On each plot, vertical dashed-lines represent each of the frequencies (1, 5 and 10 Hz) used for the continuous modification of the circle depending on the condition. Each spectrum has a frequency resolution of ~0.305 *Hz*.

**

**

**Supplementary Figure 4 Distributional and temporal statistics of circle size variations generated for the current study compared to those derived from pilot’s participant alpha power.** Each plot provides the distribution of one descriptive statistic (**A-C**: variance; **D-F**: skewness; **G-I**: kurtosis; **J-L**: lag-1 autocorrelation) for each frequency at which the circle was modified (respectively: 1 Hz, 5 Hz, and 10 Hz) and for the pilot and the current studies (x-axis; red: pilot; green: current). Each statistic was computed on the circle size variations of each participant over 60-second trials.


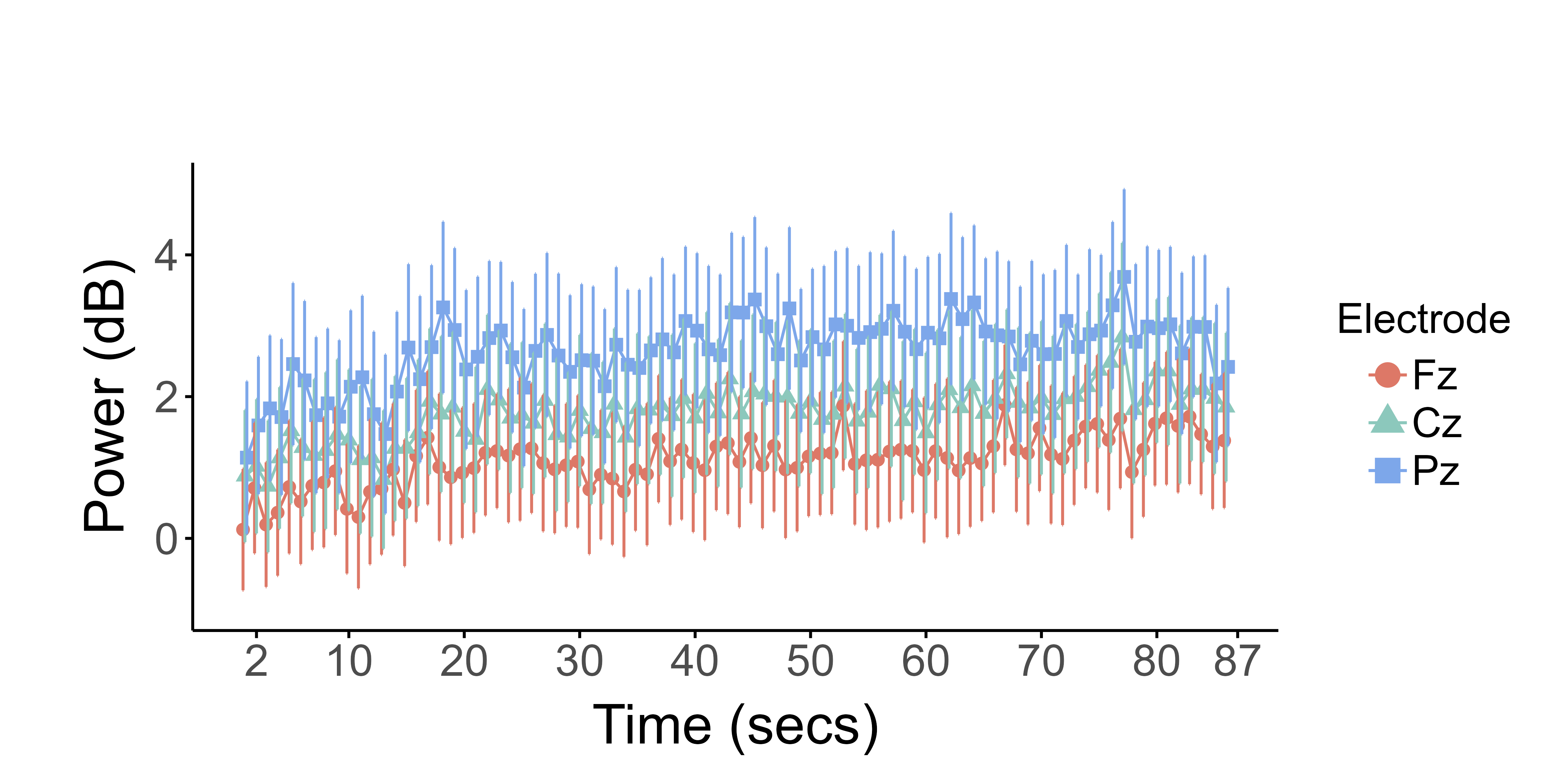
 **Supplementary Figure 5 Averaged within-trial evolution of alpha band (8-12 Hz) power in the pilot experiment.** Alpha power estimates were obtained through a time-frequency analysis performed on each trial, electrode, and subject filtered data, using a symmetric Hann window of 500 samples (50% overlap). Each line point represents the alpha spectral power averaged across the group- and trial-levels. Error bars indicate 95% confidence intervals.


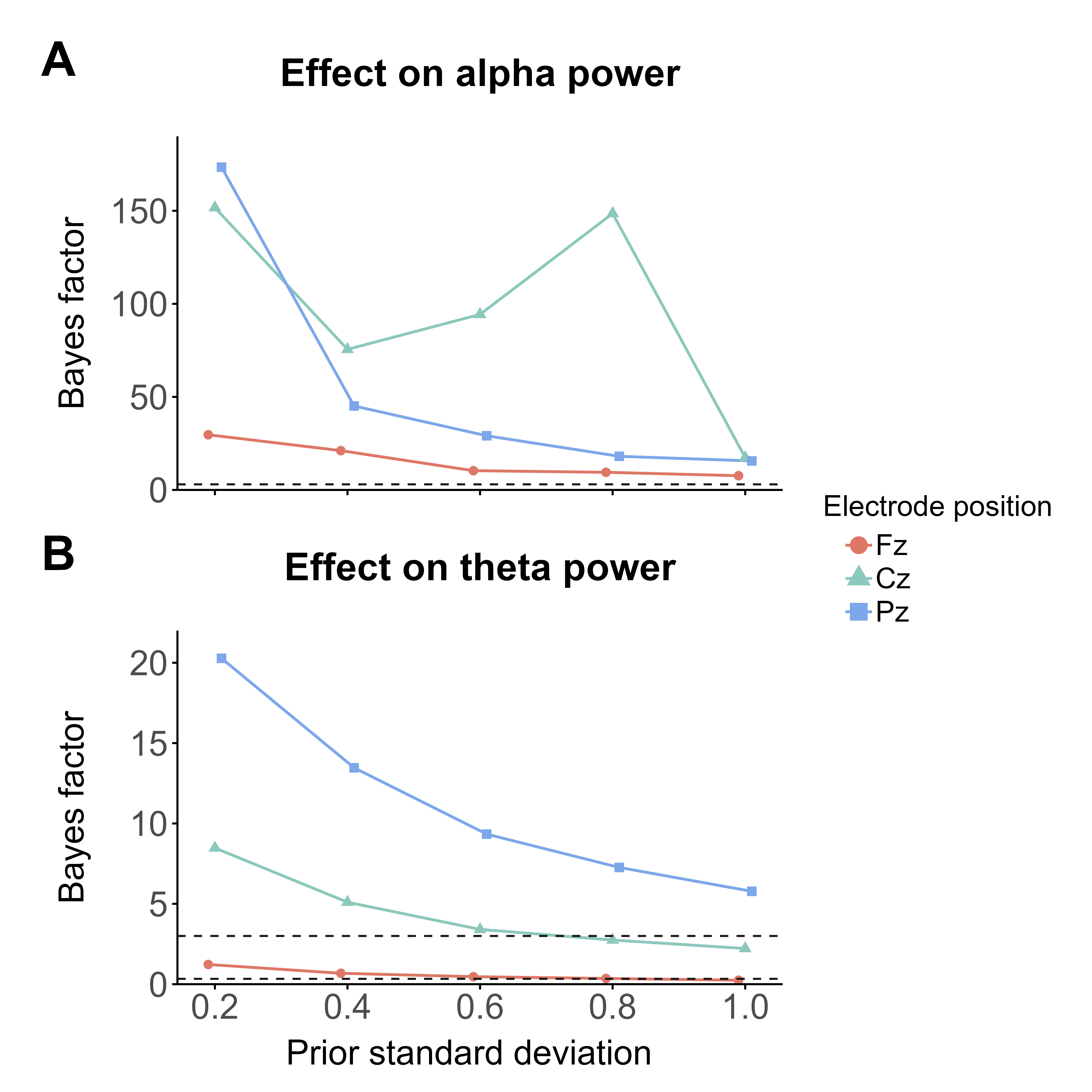
 **Supplementary Figure 6 Bayes factor in function of hypothesised effect size (sensitivity analysis).** (**A**) Evidence in favour on an effect of trial repetition on the alpha band (8-12 *Hz*) spectral power. (**B**) Evidence in favour of an effect of the continuous modification of the circle size on the theta band (4-8 *Hz*) spectral power. For both panels, each line point represents the mean of the *BF_10_* in favour of the alternative hypothesis (relative to the null) obtained across five computation of the same model (to ensure its stability). The x-axis refers to the standard deviation used to define the prior distribution (mean of 0) of the corresponding effect size.
